# Control of Bacterial Virulence through the Peptide Signature of the Habitat

**DOI:** 10.1016/j.celrep.2019.01.073

**Published:** 2019-02-12

**Authors:** Emilia Krypotou, Mariela Scortti, Christin Grundström, Melanie Oelker, Ben F. Luisi, A. Elisabeth Sauer-Eriksson, José Vázquez-Boland

**Affiliations:** 1 Microbial Pathogenesis Group, Infection Medicine, Edinburgh Medical School (Biomedical Sciences) and The Roslin Institute, University of Edinburgh, Edinburgh EH16 4SB, UK; 2 Department of Biochemistry, University of Cambridge, Cambridge CB2 1GA, UK; 3 Department of Chemistry and Umeå Centre for Microbial Research, Umeå University, 901 87 Umeå, Sweden

**Keywords:** *Listeria monocytogenes*, *Listeria* virulence regulation, PrfA allosteric regulation, environmental control of bacterial virulence, virulence regulation by nutritional peptides, Opp transport system, transcription factor regulation by peptides, PrfA-peptide 3D structure, PrfA-glutathione regulation

## Abstract

To optimize fitness, pathogens selectively activate their virulence program upon host entry. Here, we report that the facultative intracellular bacterium *Listeria monocytogenes* exploits exogenous oligopeptides, a ubiquitous organic N source, to sense the environment and control the activity of its virulence transcriptional activator, PrfA. Using a genetic screen in adsorbent-treated (PrfA-inducing) medium, we found that PrfA is functionally regulated by the balance between activating and inhibitory nutritional peptides scavenged via the Opp transport system. Activating peptides provide essential cysteine precursor for the PrfA-inducing cofactor glutathione (GSH). Non-cysteine-containing peptides cause promiscuous PrfA inhibition. Biophysical and co-crystallization studies reveal that peptides inhibit PrfA through steric blockade of the GSH binding site, a regulation mechanism directly linking bacterial virulence and metabolism. *L. monocytogenes* mutant analysis in macrophages and our functional data support a model in which changes in the balance of antagonistic Opp-imported oligopeptides promote PrfA induction intracellularly and PrfA repression outside the host.

## Highlights

• *Listeria* PrfA virulence regulation is controlled by antagonistic nutritional peptides
• Opp-imported peptides regulate PrfA upstream of the activating cofactor GSH
• PrfA is activated by peptides that provide essential cysteine for GSH biosynthesis
• Blockade of PrfA’s GSH binding site by peptides inhibits virulence gene activation

## Graphical Abstract

**Figure:**
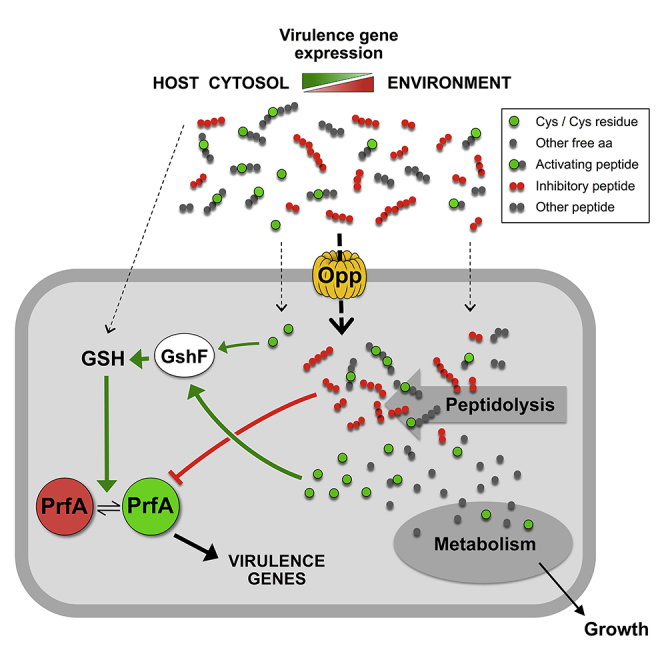

## Teaser

We identify a major control mechanism of *Listeria* virulence based on antagonistic regulation by environmental peptides. Activity levels of the virulence regulator PrfA depend on the net balance between the rates of synthesis of the PrfA-activating cofactor GSH from exogenous peptide-derived cysteine and of direct, promiscuous PrfA inhibition by non-cysteine-containing peptides.

## Introduction

*Listeria monocytogenes,* the causative agent of foodborne listeriosis, is a paradigmatic example of a pathogen exerting tight control over its virulence genes (Freitag et al., 2009). This ubiquitous gram-positive bacterium uses a set of nine virulence factors to promote host cell invasion (InlA, InlB), phagosomal escape (*hly-*encoded LLO, PlcA, and PlcB), rapid cytosolic replication (Hpt), and cell-to-cell spread (ActA, InlC) (Hamon et al., 2006). Their expression is activated during cell infection (Chatterjee et al., 2006, Shetron-Rama et al., 2002) and depends on PrfA (Mengaud et al., 1991), a transcription factor of the Crp/Fnr family (Scortti et al., 2007). PrfA is essential for pathogenesis (Chakraborty et al., 1992) but is equally important for preventing the cost of unneeded virulence factors in the environmental reservoir (Vasanthakrishnan et al., 2015).

PrfA regulation operates through control of (1) PrfA abundance, exerted at both the transcriptional and translational levels and involving positive autoregulation of the *prfA* gene, and (2) PrfA activity, via cofactor-mediated allosteric shift between low- (“Off”) and high- (“On”) activity states (reviewed in Scortti et al. [2007]). The latter is thought to play a key role in the strong PrfA induction observed during intracellular infection (Deshayes et al., 2012). Single amino acid substitutions, called PrfA^∗^ mutations, lock PrfA in “On” conformation with increased DNA-binding activity (Eiting et al., 2005, Vega et al., 1998), causing constitutive activation of virulence genes to high, “infection-like” levels (Ripio et al., 1997b, Shetron-Rama et al., 2003, Vega et al., 2004). Recently, a genetic screen in macrophages found that the thiol-redox buffer glutathione (GSH, γ-L-Glutamyl-L-cysteinylglycine) (Loi et al., 2015), endogenously produced by the listerial GshF enzyme (Gopal et al., 2005), was required to promote PrfA activation (Reniere et al., 2015). Exogenous GSH had a similar PrfA-inducing effect *in vitro* in synthetic medium (Portman et al., 2017). Co-crystallization studies showed that GSH binds in a large tunnel between PrfA’s N-terminal and C-terminal domains, priming PrfA for productive interaction with the target DNA (Hall et al., 2016). While GSH is required for full PrfA induction and intracellular proliferation (Gopal et al., 2005, Reniere et al., 2015), how GSH-dependent PrfA activity is regulated remains to be clarified.

A combination of environmental and endogenous cues converge on PrfA to modulate virulence expression. These include temperature via an RNA thermoswitch that controls *prfA* translation (Johansson et al., 2002), stress signals via a SigB-regulated *prfA* promoter (Nadon et al., 2002), a reducing environment (Portman et al., 2017), and metabolic signals, including carbon-source nutrition (Joseph et al., 2008, Milenbachs et al., 1997, Ripio et al., 1997a) or amino acid availability (Haber et al., 2017, Lobel et al., 2015, Xayarath et al., 2009) through as yet not fully understood mechanisms. In addition to the intracellular milieu and GSH, treating the growth medium with activated charcoal also causes strong PrfA induction (Ripio et al., 1996, Milohanic et al., 2003). This phenomenon is observed in complex media, such as brain-heart infusion (BHI), where PrfA-dependent expression is very weak at 37°C. Adsorbent resins, such as Amberlite XAD4, have the same effect, suggesting that the mechanism involves the sequestration of PrfA inhibitory substances (Ermolaeva et al., 2004).

In this study, we performed a transposon screen to characterize the molecular basis of the intriguing effect of adsorbents on listerial virulence expression. We show that this effect depends on a functional Opp oligopeptide transporter, which allows *L. monocytogenes* to control PrfA-GSH regulation according to the “peptide signature” of the bacterial habitat.

## Results

### Genetic Screen for Amberlite XAD4 Non-activable Mutants

**Figure 1:**
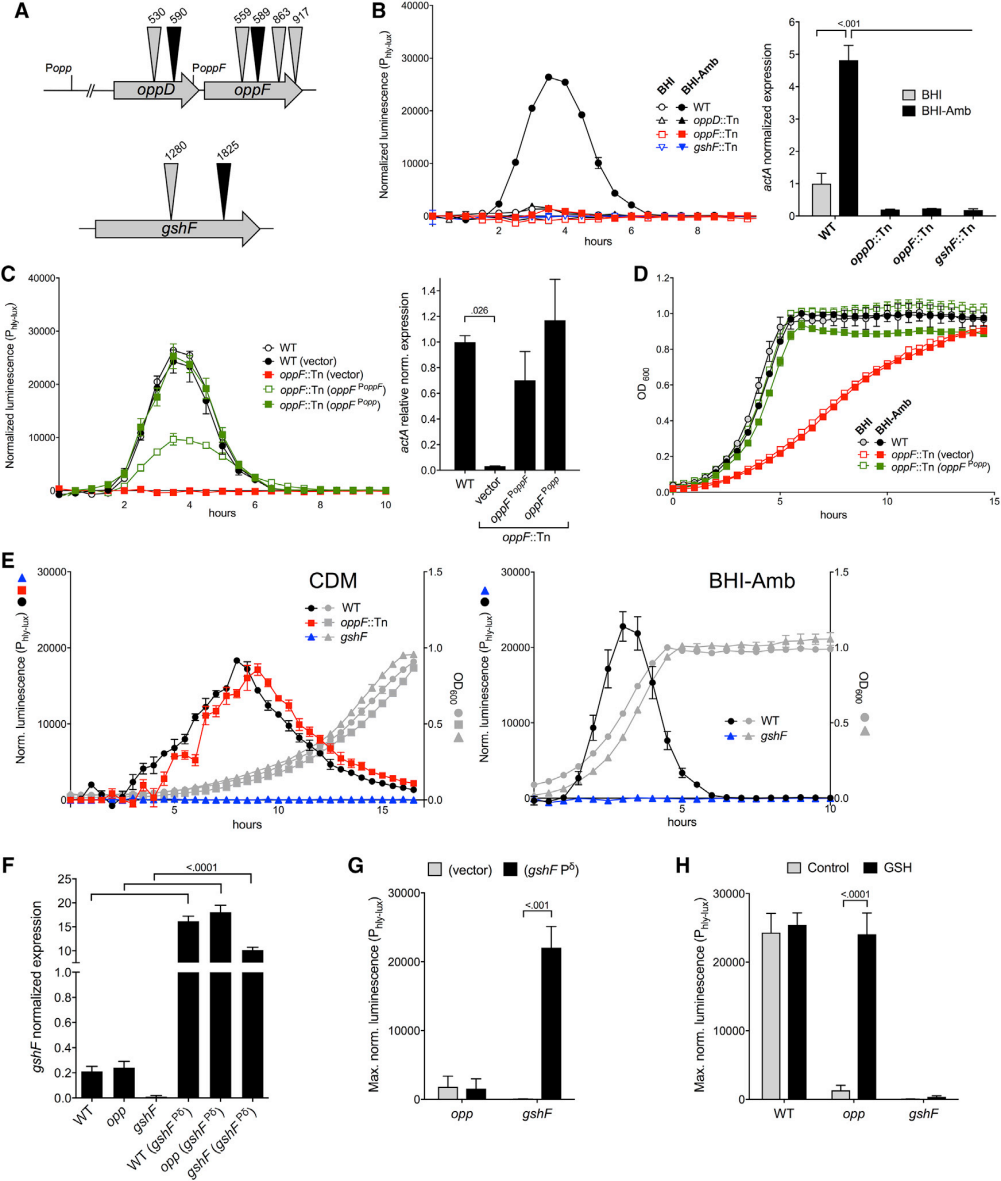
Characterization of Tn Mutants (A) Transposon insertions. In black, those selected for detailed analysis. Coordinates from the start of an ORF. Promoters are as characterized in Wurtzel et al. (2012). (B) PrfA-dependent expression of wild-type P14 (WT) and *opp* and *gshF* Tn mutants using P_hly-lux_ reporter (left) and *actA* transcription analysis by qRT-PCR (right, data expressed as relative values to WT). (C) Complementation of *oppF589*::Tn in BHI-Amb with *oppF* expressed from its own promoter (*oppF*^PoppF^) or *opp* operon promoter (*oppF*^Popp^), or with empty vector. (D) Growth curves of WT and *oppF589*::Tn complemented with *oppF* or empty vector. (E) Opp^–^ mutant exhibits wild-type (PrfA^+^) phenotype in CDM. Left: P_hly-lux_ reporter normalized luminescence and growth curves (OD_600_) of WT, *oppF*::Tn, and isogenic Δ*gshF* mutant in CDM. Right: phenotype of Δ*gshF* in BHI-Amb. (F) Overexpression of *gshF* under the strong Pδ promoter (de la Hoz et al., 2000) (*gshF*^Pδ^) in WT, *oppF589*::Tn (*opp*), and Δ*gshF. gshF* transcription by qRT-PCR in BHI-Amb. Non-complemented bacteria contain an empty vector. (G) *gshF* overexpression does not rescue the PrfA^–^ phenotype of *opp* mutant in BHI-Amb. P_hly-lux_ maximum normalized luminescence. (H) Rescue of *opp* mutant by exogenous GSH (1 mM) in BHI-Amb. Note that 1 mM exogenous GSH did not revert the PrfA^–^ phenotype in Δ*gshF* (even at a concentration of 8 mM; data not shown). This may reflect that, in BHI-Amb, exogenous GSH is insufficient for the intrabacterial GSH concentration reaching a threshold for normal PrfA activity in the absence of an endogenous (GshF-derived; Gopal et al., 2005) GSH pool. Data in (B) and (C) left, (D), and (E) are mean ± SEM from a representative experiment of at least three biological replicates; in (B) and (C) right, and (F)–(H), means ± SEM of three independent experiments, each in tripiclate. Significant p values are indicated (B, right; C, right; and F, one-way ANOVA; G and H, two-way ANOVA). See also Figures S1 and S2.

A *himar1* transposon (Tn) library was constructed in *L. monocytogenes* P14-P_hly-lux_, a wild-type serovar 4b isolate carrying a chromosomally integrated *luxABCDE* reporter under the control of the PrfA-regulated *hly* promoter (Bron et al., 2006). “Non-activable” (PrfA^–^) Tn mutants were selected in Amberlite XAD4-treated BHI (BHI-Amb) by exploiting the ability of the PrfA-regulated organophosphate permease Hpt to confer susceptibility to the antibiotic fosfomycin (Scortti et al., 2006) (see STAR Methods). Apart from *prfA* and *hpt*, two other loci were redundantly targeted upon screening ≈500 fosfomycin resistant mutants (Figure 1A): *gshF* encoding the listerial GSH synthase, the inactivation of which was previously shown to result in reduced PrfA-dependent expression (Reniere et al., 2015); and *oppDF* encoding the ATPase subunits of the Opp oligopeptide transport system (Borezee et al., 2000).

The *oppD/F*::Tn mutants exhibited similar phenotype to the *gshF*::Tn mutants, characterized by a pleiotropic PrfA-regulated gene activation defect in BHI-Amb as determined using reporter gene tests (Figures S1A–S1C) and promoter activation/gene expression analyses (Figure 1B). Knockout mutagenesis of *oppD* and *oppF* recapitulated the PrfA^–^ phenotype (Figure S1D). Complementation of one of the Tn mutants selected for further characterization (*oppF589*; Figure 1A) rescued the parental wild-type PrfA^+^ phenotype (Figure 1C). This identified the *opp* locus as potentially involved in PrfA regulation.

### Link between Opp Peptide Transport and PrfA Regulation

*oppF*::Tn (all *opp* Tn mutants) showed impaired growth in BHI and acquired resistance to bialaphos, a toxic tripeptide that bacteria take up through Opp permeases (Borezee et al., 2000). Complementation rescued both phenotypes (Figures 1D and S1E), confirming that the *oppF* mutation disabled Opp function. For simplicity, *oppF*::Tn is henceforth designated as *opp* (or Opp^–^) mutant. As expected, *opp* bacteria showed wild-type growth in chemically defined medium (CDM) only containing free amino acids as proteinogenic N (Figure 1E, left). Notably, in CDM, the *opp* mutant also exhibited a PrfA^+^ phenotype equivalent to that of the wild-type parent in BHI-Amb (Figure 1E), whereas Amberlite XAD4 has no effect on wild-type *L. monocytogenes* (Figure S2). These data implied that the adsorbent removes some critical Opp-transported BHI component(s), presumably of peptide nature, which affect(s) PrfA regulation.

Since growth in CDM rescued the *opp* mutant, and a Δ*gshF* mutant constructed in P14 exhibited PrfA^–^ phenotype in both CDM and BHI-Amb (Figure 1E), *gshF* is clearly downstream of *opp* and/or dominant in the PrfA regulation pathway. Transcription analysis excluded that the PrfA^–^ phenotype of the *opp* mutant in BHI-Amb was due to reduced *gshF* expression (Figure 1F). In addition, overexpression of *gshF* under the control of a strong promoter (*P*δ; de la Hoz et al., 2000) (Figure 1F) did not reverse the PrfA^–^ phenotype of *opp* bacteria in BHI-Amb, while it successfully complemented the Δ*gshF* mutation (Figure 1G). However, exogenous addition of 1 mM GSH fully restored the parental PrfA^+^ phenotype in the *opp* mutant (Figure 1H). Thus, when Opp function is affected, the limiting factor for PrfA activation does not seem to be the levels of *gshF* expression but, critically, the amounts of its biosynthetic product, GSH. Overall, these results suggested that an Opp-transported BHI component controls the synthesis or availability of endogenous GSH for PrfA activation.

### Cysteine as Part of an Oligopeptide Mediates Opp-Dependent PrfA Upregulation

**Figure 2:**
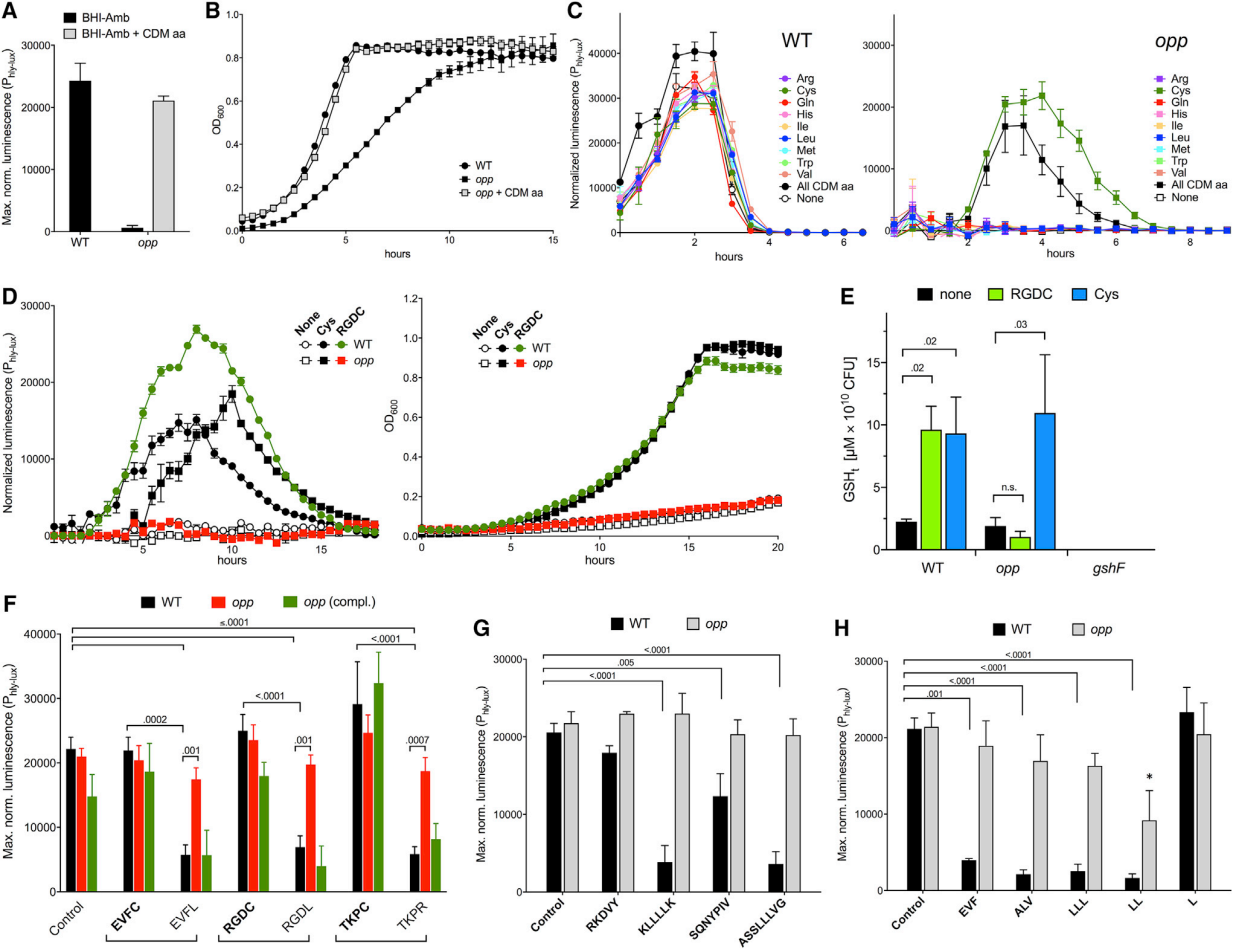
PrfA Regulation by Cys- and Non-Cys-Containing Peptides (A) Rescue of *opp* mutant by supplementation of BHI-Amb with CDM amino acids (same final concentration). Expression level of WT shown as reference. Mean ± SEM of two triplicate experiments. (B) Representative growth curves from (A). Supplementation of BHI-Amb (and BHI, not shown) with CDM amino acids restores WT growth in *opp* mutant. (C) Rescue of *opp* mutant by L-cysteine. BHI-Amb was supplemented with the same concentration of CDM amino acids added in a mix or individually. (D) Opp-dependent PrfA induction by Cys-containing oligopeptide. P_hly-lux_ expression (left) and growth (right) in CDM (without Cys) supplemented with 0.8 mM Cys or 0.32 mM RGDC peptide. Data in (C) and (D) are means ± SEM of a representative triplicate experiment. (E) Opp-dependent GSH synthesis. Total GSH (GSHt) was determined in wild-type and *opp L. monocytogenes* grown in CDM containing 0.2 mM Cys and supplemented with 1 mM RGDC peptide or free Cys. Δ*gshF*, negative control. GSHt expressed as μM per 10^10^ CFU. Mean ± SEM of three experiments in duplicate. (F–H) Opp-dependent PrfA inhibition by non-Cys peptides in CDM. Means ± SEM of three triplicate experiments. Statistically significant p values are indicated (two-way ANOVA). (F) P_hly-lux_ expression in WT, *opp* mutant and complemented *opp* mutant (compl.) in response to 1 mM synthetic tetrapeptides containing or not containing Cys. Control, no peptide. *opp* mutant carries empty vector. (G) Effect of several 5- to 8-mer non-Cys synthetic peptides on WT and *opp* mutant. (H) Effect of several tripeptides, Leu dipeptide, and 1 mM free L-Leu. Note the partial Opp-independent inhibition by LL, suggesting alternate import by other (dipeptide) transporter(s) (Monnet, 2003, Wouters et al., 2005). Asterisk indicates p = 0.009 relative to *opp* mutant in control conditions. See also Figures S3 and S4.

Adding all CDM amino acids to BHI-Amb rescued the wild-type PrfA^+^ (and growth) phenotype in the *opp* mutant (Figures 2A and 2B). We traced the effect to L-cysteine (Cys) (Figure 2C). Although Cys is an essential amino acid for *L. monocytogenes* (Tsai and Hodgson, 2003; Figure S3A), dose-dependent PrfA induction was observed in CDM for both wild-type and *opp* bacteria once the minimum concentration for eugonic growth (≈0.2 mM) had been reached (Figure S3B). Since adding Cys to BHI-Amb recapitulated the functional complementation by GSH, and Cys is an essential rate-limiting precursor for GSH biosynthesis (Loi et al., 2015, Lu, 2009), we reasoned that the PrfA^–^ phenotype of the *opp* mutant could result from an inability to incorporate Cys in oligopeptide form. Confirming this, like free Cys, a Cys-containing tetrapeptide (RGDC) promoted growth and PrfA-dependent expression in wild-type *L. monocytogenes*, but not in the *opp* mutant (Figures 2D, S3C, and S3D). That the Δ*gshF* mutant was not rescued in (Cys-replete) CDM (Figure 1E, left) rules out that Cys acts as a direct PrfA activator. Thus, the PrfA^–^ phenotype of Opp^–^ *L. monocytogenes* in BHI-Amb is most likely explained by an inability to incorporate Cys-containing peptides for endogenous (GshF-mediated) biosynthesis of the PrfA-activating cofactor GSH in Cys-limiting conditions. Total GSH determinations in bacteria grown in CDM with limiting (0.2 mM) Cys (Figure S3AB) confirmed that both RDGC peptide and free Cys were required for synthesis of the PrfA cofactor, the former in an Opp-dependent manner (Figure 2E). In addition to an essential GSH building block, the amino acid Cys could act as a thiol donor (Ohtsu et al., 2010), potentially contributing to a reducing environment important for PrfA activation (Portman et al., 2017).

### PrfA Repression by Non-Cys-Containing Peptides

We compared the effect of Cys-containing synthetic oligopeptides (Cys-peptides) transported by Opp (RGDC, EVFC, TKPC; Figure S4) and versions thereof with Cys replaced by another residue (RGDL, EVFL, TKPR). Regular CDM (0.8 mM Cys) was used to ensure normal growth in the absence of Cys-peptides. While 1 mM Cys-peptide did not alter (or increased) P_hly-lux_ expression, equivalent amounts of the corresponding non-Cys-peptides caused significant Opp-dependent PrfA downregulation (69%–74%, p < 0.001) (Figure 2F). TKPR is aka tuftsin, a mamalian immunomodulatory tetrapeptide from the Fc domain of immunoglobulin G (IgG) (Wu et al., 2012). A listerial derived octapeptide, ASSLLLVG (putative peptide pheromone pPplA; Xayarath et al., 2015), also caused comparable Opp-dependent repression (88%) (Figure 2G). Of three known > 5-mer listerial Opp substrates (Borezee et al., 2000, Whiteley et al., 2017), two were significantly inhibitory (KLLLLK 96%, SQNPYPIV 59%, RKDVY no effect) (Figure 2G). Tripeptides also caused Opp-dependent PrfA downregulation, as illustrated with EVF (truncated derivative of EVFC/L, 81%), ALV (90%) or LLL (3-mer peptide of Leu used to replace the Cys residue in two of the above repressing tetrapeptides, 87%) (Figure 2H). A Leu dipeptide caused the same strong inhibition as LLL (93%). However, equivalent molar amounts of free Leu were not inhibitory (p > 0.999), indicating that PrfA repression is specifically linked to the peptide form of the amino acid (Figure 2H). Other tested dipeptides showed different degrees of PrfA repressing activity (AF 93%, AL 75%, FV 45%, AG 0%).

Overall, our data show that in PrfA-permissive (Cys-replete) conditions, many tested non-Cys peptides, including host- or bacteria-derived peptides, inhibit PrfA.

### Balance of Inducing and Inhibitory Peptides Controls PrfA

**Figure 3:**
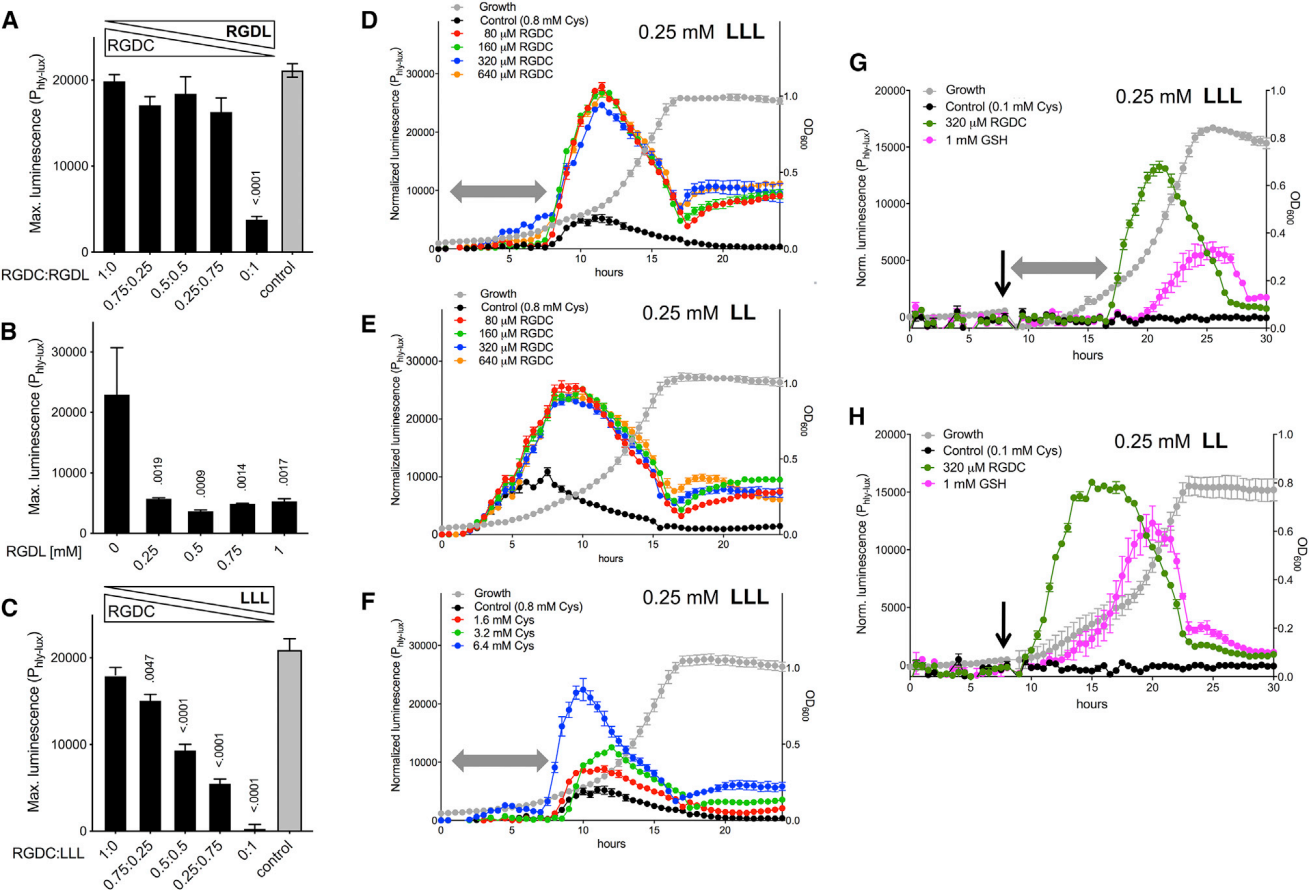
Antagonistic Control by PrfA-Inducing and PrfA-Repressing Peptides (A–C) P_hly-lux_ maximum normalized luminescence of WT in CDM containing mixtures of inducing RGDC peptide and cognate repressing RGDL peptide (A), same experiment without RGDC peptide (B), or RGDL peptide replaced by strongly repressing LLL peptide (C). Final peptide concentration, 1 mM; control, no peptide. Mean ± SEM of three triplicate experiments. p values relative to first column are shown (one-way ANOVA). (D–H) Reversal of Leu peptide-mediated PrfA repression by Cys-peptide (D, E, G, H), free Cys (F), or GSH (G, H). Experiments performed in CDM containing limiting Cys (0.1 mM). Normalized luminescence of WT along the bacterial growth curve (average OD_600_ values in gray). Gray double arrows and downward-pointing arrows indicate the expression delay caused by LLL, but not LL, peptide, and time of addition of RGDC peptide or GSH, respectively. Note in (D)–(H) the gradual decline of the expression signal until the end of the exponential growth phase, likely reflecting progressive exhaustion of the PrfA-stimulating input (and, eventually, accumulation of bacteria-derived PrfA repressor products in the medium (Ermolaeva et al., 2004). Data in (D)–(H) are means ± SEM of a representative triplicate experiment.

Next, we analyzed the combined effect of PrfA-inducing and inhibitory peptides. In RGDC/RGDL titrations, the Cys-peptide was clearly dominant at all tested RGDL concentrations (Figure 3A), while maximal inhibition was observed when RGDC was omitted (Figure 3B). In contrast, RGDC/LLL titrations resulted in a linear repression response as the LLL concentration increased (Figure 3C). These data show that different inhibitory peptides differ in the ability to counteract the PrfA-stimulating effect of Cys-peptides.

We also tested the effect of increasing RGDC concentrations against a fixed “non-saturating” amount (0.25 mM) of the strongly repressing Leu di- and tripeptides. Even at the low concentration of 80 μM, the RGDC peptide completely cancelled LL/LLL-mediated repression (Figures 3D and 3E). Comparatively larger amounts of free Cys (6.4 mM) were required to achieve the same effect (Figure 3F). When 320 μM RGDC or 1 mM GSH were added after several hours of growth in CDM containing limiting Cys (0.1 mM, sufficient to promote growth but not PrfA-dependent expression), the Cys-peptide was again more effective in countering LL/LLL-mediated repression (Figures 3G and 3H). Thus, peptide-mediated PrfA inhibition is more efficiently reversed by Cys-peptides than free Cys or, indeed, exogenous GSH, underscoring the importance of Opp in PrfA regulation.

Interestingly, with no differences in the bacterial growth dynamics, a protracted repression was observed with LLL, but not LL, until RGDC, Cys, or GSH exhibited their PrfA-stimulatory effect (Figures 3D–3H). This is likely due to release of repressing LL dipeptide intermediate during the metabolic breakdown of LLL into non-repressing free Leu (Figure 2H).

Collectively, our results indicate that PrfA induction levels depend on the balance of inhibitory and inducing oligopeptide inputs from the medium, and that the stoichiometry and dynamics of this balance is critically affected by the composition of the peptide mixture.

### Opp Is Required for Early PrfA Induction within Host Cells

**Figure 4:**
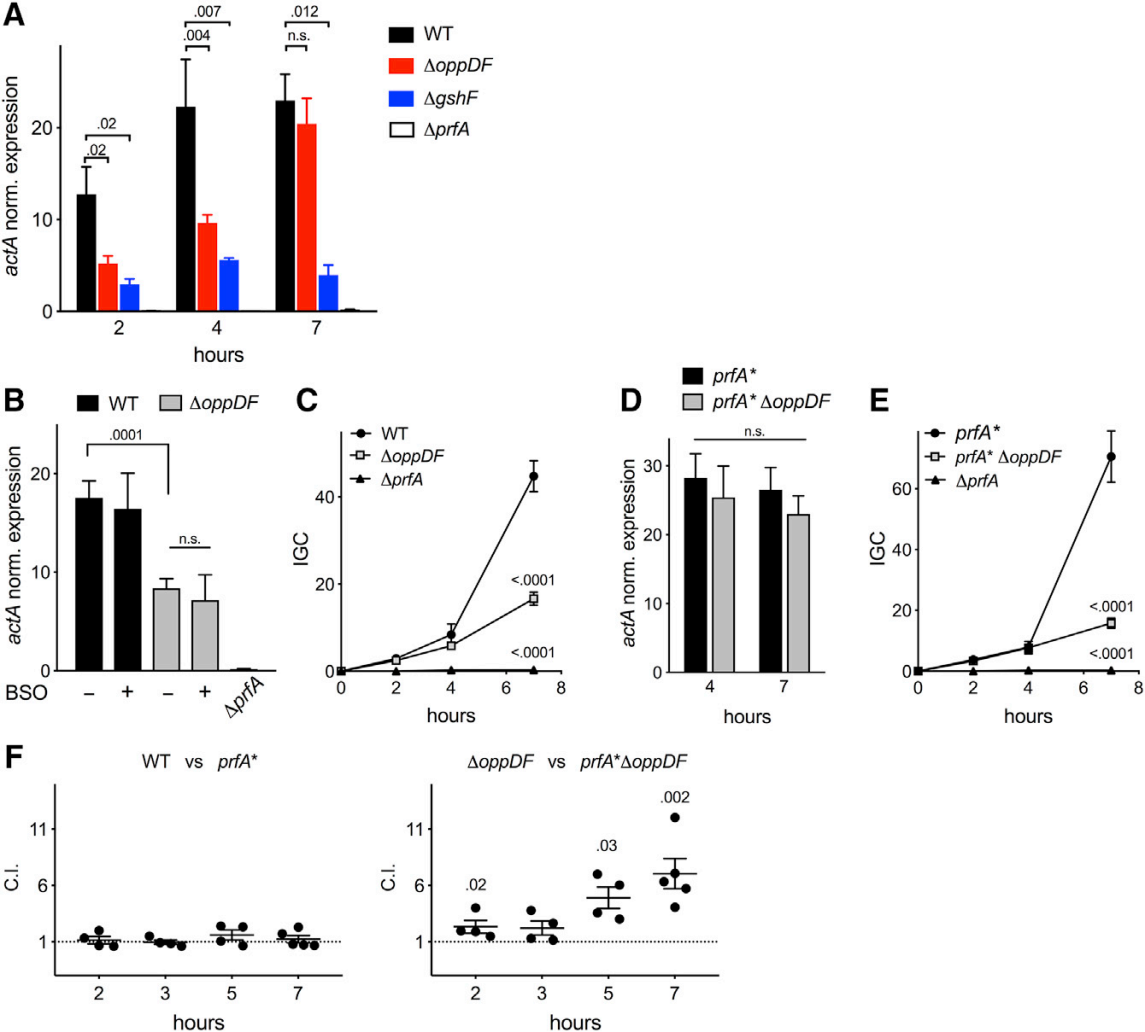
Opp-Dependent PrfA Activation and Replication within Host Cells (A) *actA* transcription analysis by qRT-PCR of WT, Δ*oppDF*, and (control) Δ*gshF* and Δ*prfA* derivatives in J774A.1 mouse macrophages at *t* = 2, 4, and 7 h after infection. (B) *actA* transcription analysis of WT and Δ*oppDF* mutant in J774A.1 cells treated with the GSH-depleting drug buthionine sulfoximine (BSO) at *t* = 4 h post-infection. Prior to infection, cells were incubated in normal (–) or Cys-free medium followed by BSO treatment (+). (C) Intracellular replication of WT and Δ*oppDF* in J774A.1 cells. Data are expressed as the normalized intracellular growth coefficient (IGC; see STAR Methods). (D) *actA* transcription analysis of *L. monocytogenes prfA*^∗^^G145S^ and *prfA*^∗^Δ*oppDF* in J774A.1 cells. (E) Same as in (C) using bacteria with *prfA*^∗^ allele. (F) Competition assay in J774A.1 macrophages between *L. monocytogenes* P14 with wild-type *prfA* allele (WT, Δ*oppDF*) and constitutively activated *prfA*^∗^ allele (*prfA*^∗^, *prfA*^∗^Δ*oppDF*). Left: comparison in Opp^+^ background (Opp-dependent PrfA activation enabled). Right: comparison in Opp^–^ background (Opp-dependent PrfA activation disabled). The bacteria used in these experiments do not contain the *luxABCDE* reporter. C.I., competitive index; values > 1 indicate competitive advantage for *prfA*^∗^ bacteria. Data are means ± SEM of at least two (A, B, D), three (C and E), or four (F) triplicate experiments. Relevant statistical comparisons are indicated. Two-way ANOVA except one-way ANOVA in (D) and one-sample Student’s t test (hypothetical value of 1, two-tails) in (F). See also Figures S5 and S6.

We examined whether the Opp transport system plays any role in intracellular PrfA activation in infected J774 mouse macrophages. An *oppDF* deletion mutant was used to avoid potential problems of transposon instability in the harsher intracellular conditions. P14Δ*oppDF* exhibited PrfA and oligopeptide transport phenotypes indistinguishable from those of the Tn mutants (Figures S1F and S1G). Intracellular PrfA induction, as monitored by *actA* transcription, was significantly reduced (≈60%) in Δ*oppDF* at *t* = 2 h and 4 h compared to wild-type (Figure 4A). The induction defect was similar (*t* = 2 h, p = 0.53), or marginally less pronounced (*t* = 4 h, p = 0.04), to that of control PrfA activation-deficient Δ*gshF* (Reniere et al., 2015). However, no differences were observed at later stages of intracellular infection (*t* = 7 h) (Figure 4A). These results indicate that Opp is required for early intracellular PrfA activation, presumably by permitting the incorporation of Cys in peptide form according to our *in vitro* functional data. To further document this, macrophages were deprived of Cys and then pre-treated with the GSH-depleting drug buthionine sulfoximine (BSO) (Reniere et al., 2015, Rouzer et al., 1981) to minimize the potential input of host cell-derived free Cys and GSH pools. In these conditions, Δ*oppDF* exhibited the same *actA* induction defect at *t* = 4, while no significant changes were observed for the wild-type (Figure 4B), consistent with the PrfA activation deficit being attributable to defective import of Cys-containing peptides.

### Nutritional versus PrfA Regulatory Roles of Opp in Virulence

Experiments with Δ*oppDF* show that listerial Opp is required for efficient growth in macrophages (Figure 4C) (Borezee et al., 2000) and full virulence in a mouse model of systemic infection (Figure S5). This could result from either defective activation of the PrfA virulence regulon (Figure 4A), or defective growth due to impaired utilization of host-derived peptides (Figures 1D, 2B, and 2D, right). To dissect this, we analyzed the intracellular phenotype of Δ*oppDF* with PrfA regulation bypassed using a *prfA*^∗^^G145S^ allele (Figure 4D). *prfA*^∗^^G145S^ bacteria overexpress the PrfA regulon without the need of adding adsorbents to the BHI (Ermolaeva et al., 2004, Ripio et al., 1996, Ripio et al., 1997b), independently or *gshF*/GSH (Reniere et al., 2015), and are largely unsusceptible to peptide-mediated regulation (Figure S6). Growth of *prfA*^∗^Δ*oppDF* was still strongly affected (Figure 4E), indicating that peptides are used as the main amino acid source intracellularly, consistent with previous data using auxotrophic mutants (Marquis et al., 1993).

To assess the impact of Opp-dependent PrfA activation, we compared the intracellular dynamics of Δ*oppDF* expressing wild-type PrfA (PrfA^WT^), which necessitates activation to promote infection (Deshayes et al., 2012), or constitutively activated PrfA^∗^. Because the strong nutritionally related proliferation defect caused by the Opp^–^ mutation could mask PrfA-related effects (see Δ*oppDF* bacteria in Figures 4C and 4E), we used a competition assay to enhance discrimination. No differences in competitive ability were observed between PrfA^WT^- and PrfA^∗^-expressing Opp-proficient bacteria, confirming that the levels of virulence gene activation are in both cases similar (Figure 4F, left). In contrast, when Opp was absent, PrfA^WT^ bacteria (requiring Opp for efficient PrfA activation; Figure 4A) were outcompeted by those with constitutively activated PrfA^∗^ (Figure 4F, right). Overall, these data identify Opp as an important listerial virulence determinant with key dual roles in N nutrition and PrfA activation within host cells.

### Peptide-Mediated Regulation Is Due to Changes in PrfA Activity

**Figure 5:**
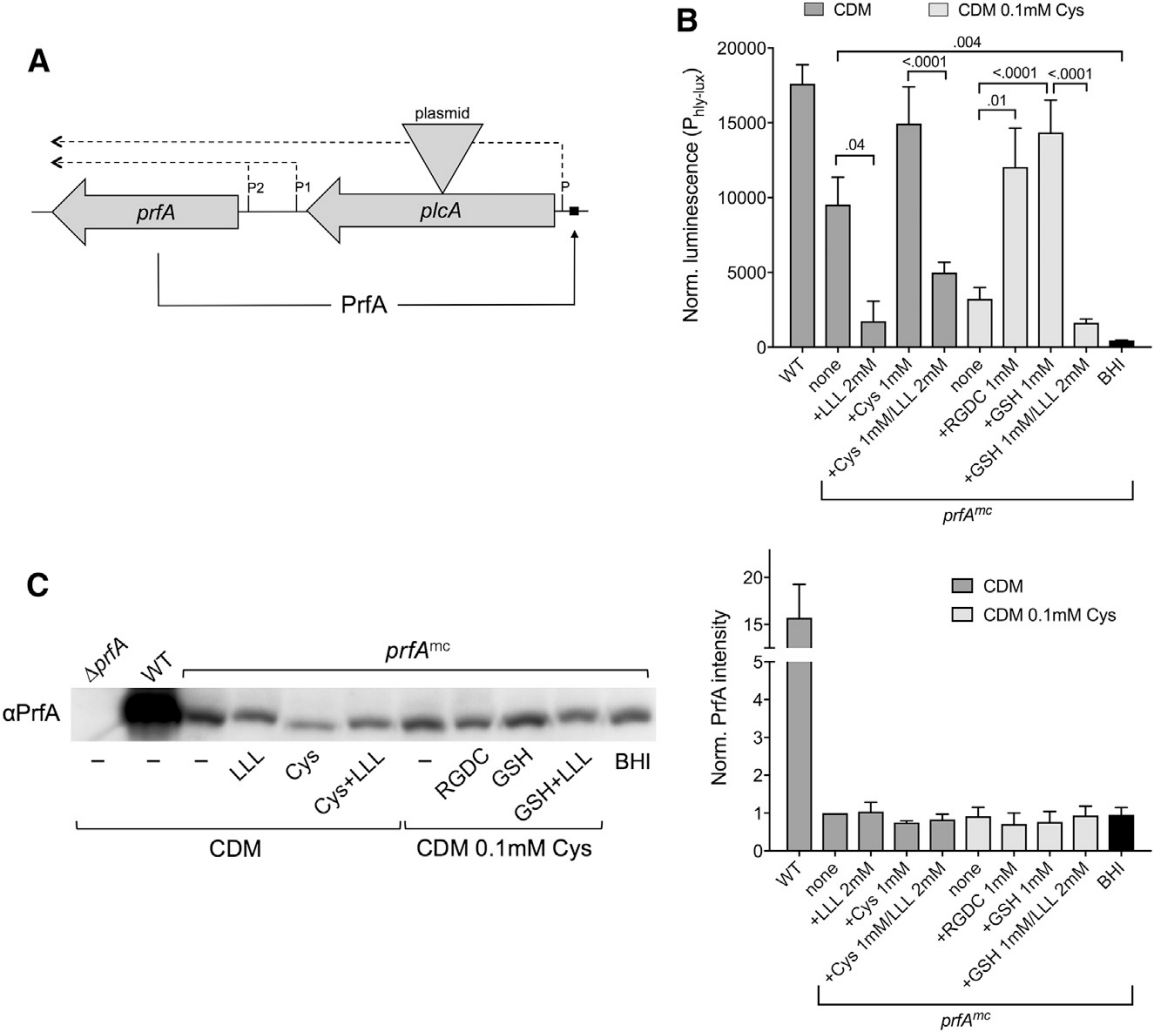
Peptide Regulatory Effects Are Due to Changes in PrfA Activity (A) Positive transcriptional autoregulation of PrfA. Disruption of the PrfA-dependent *plcA-prfA* message that drives the autoamplification loop (Mengaud et al., 1991) by insertional mutagenesis of the *plcA* gene (strain P14*prfA*^mc^). In these conditions, PrfA protein amounts only depend on the expression/translation levels of the non-PrfA-regulated monocistronic *prfA* message (reviewed in Scortti et al. [2007]). Relevant transcripts are shown; the black square is the *plcA* PrfA-box (shared with the divergently transcribed *hly* gene, which is not shown). (B) P_hly-lux_ maximum normalized luminescence of *L. monocytogenes* P14 (WT control) and P14*prfA*^mc^ derivative in conditions leading to different levels of PrfA induction. Mean ± SEM of three triplicate experiments. Relevant p values are indicated (one-way ANOVA). (C) PrfA western blot of *L. monocytogenes* cell extracts obtained in (B). Left: representative immunoblot, ≈2-3 μg of total protein in each lane. Right: PrfA quantification from densitometric scannings of the blots. Mean ± SEM of pooled cultures from experiments in (B). Values for P14*prfA*^mc^ are not significantly different (one-way ANOVA). Note the 15-fold greater PrfA protein amount in wild-type *L. monocytogenes* compared to P14*prfA*^mc^ due to the functionality of PrfA’s positive autoregulation.

To explore the mechanism behind PrfA regulation by peptides, we examined the correlation between PrfA-dependent expression and PrfA protein abundance in activating and inhibiting conditions. Since PrfA positively autoregulates its own gene (Mengaud et al., 1991) (Figure 5A), variations in PrfA activity also affect PrfA concentration (Vega et al., 1998). This problem was circumvented by disrupting the transcriptional positive feedback loop (strain P14*prfA*^mc^; Figure 5A). Even without PrfA autoregulation, P14*prfA*^mc^ showed the expected PrfA induction patterns under strongly upregulating (CDM medium supplemented with extra Cys, Cys-peptide or GSH) or downregulating (addition of repressor peptide, growth in BHI) conditions (Figure 5B). Despite the widely different expression levels, no concomitant changes in PrfA abundance were observed by western immunoblotting (Figure 5C). Thus, PrfA regulation by peptides is primarily exerted via control of PrfA protein activity, not *prfA* gene expression.

### Mechanism of Peptide-Mediated PrfA Inhibition

**Figure 6:**
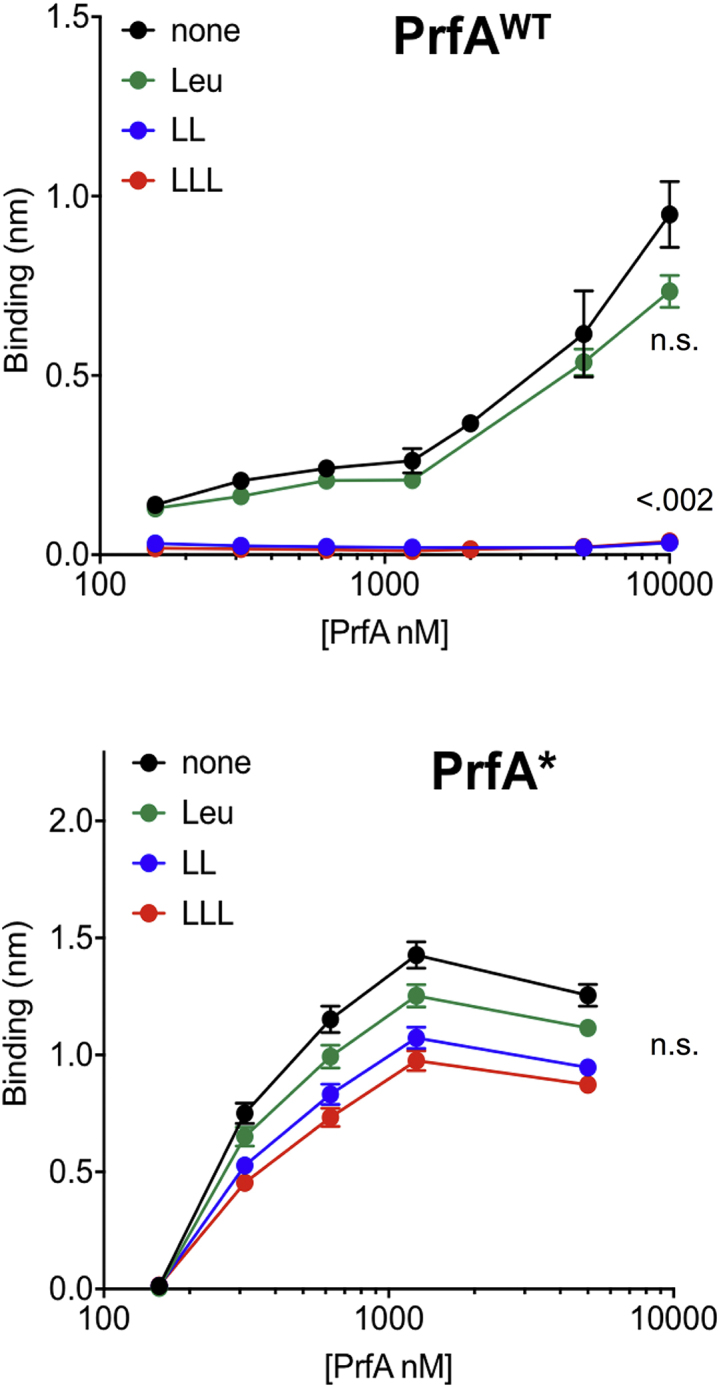
Peptide-Mediated Inhibition of PrfA-DNA Binding Effect of inhibitory Leu peptides and free Leu on PrfA binding to the P*plcA/hly* PrfA box measured by biolayer interferometry. Top: data for PrfA^WT.^ showing strong PrfA-DNA binding inhibition by the LL and LLL peptides but not free Leu. Bottom: data for constitutively activated PrfA^∗^ showing unsusceptibility to inhibition (consistent with functional data in Figure S6). Graphs represent the binding signal plotted against PrfA concentration. Mean ± SEM of at least two experiments. Relevant statistical comparisons are indicated (one-way ANOVA). See also Figures S6 and S7.

While the effect of Cys/Cys-peptides is explained by their essential role in the synthesis of the PrfA cofactor GSH, different mechanisms may underlie peptide-mediated inhibition of PrfA activity. We tested the simplest scenario, i.e., direct binding to PrfA. Weak, albeit reproducible, increases in the melting temperature (*T*_m_) of purified PrfA, indicative of potential ligand-mediated protein stabilization, were observed for the strongly repressing LL and LLL peptides in thermal shift assays (Renaud et al., 2016) (Figures S7A and S7B). Isothermal titration calorimetry (ITC) assays (Renaud et al., 2016) demonstrated that both peptides have high affinity for PrfA (K_d_ ≈25 μM), while no binding was detected for the non-inhibitory free Leu (Figure S7C). Biolayer interferometry assays (Citartan et al., 2013) using a biotinylated oligonucleotide containing the PrfA box of the P*plcA*/P*hly* promoters demonstrated that the Leu peptides, but not free Leu, strongly inhibit PrfA-DNA binding (Figure 6).

**Figure 7:**
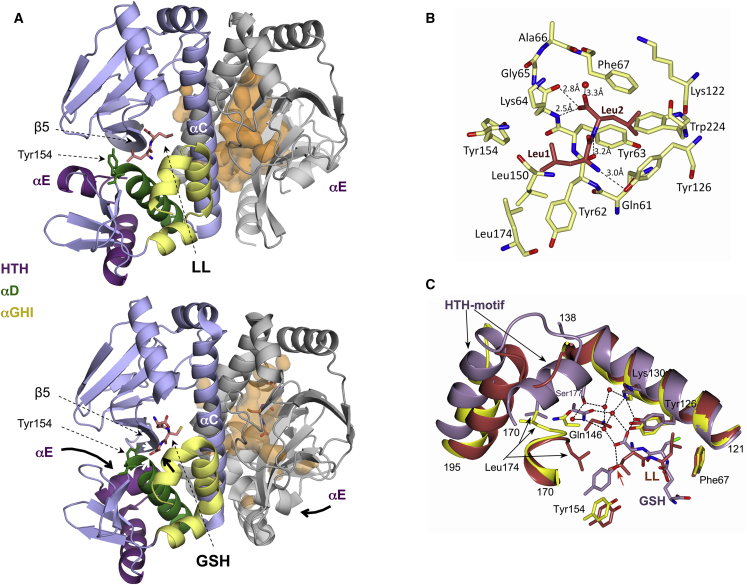
Structure of PrfA in Complex with LL Dipeptide (A) Ribbon representation of PrfA homodimer showing the binding sites of LL (top) and GSH (bottom) at the interdomain tunnel. Monomers A and B are colored in blue and gray, respectively, and the ligands are in stick representation (with C atoms in salmon color). Specific features of the C-terminal DNA-binding domain are indicated in monomer A, including Tyr154 (αD) involved in PrfA’s GSH-mediated activation and LL-mediated inhibition. Critical movements in GSH-mediated PrfA activation, which lead to the partial collapse of the tunnel and repositioning of HTH’s αE—prevented by LL binding—are indicated in the bottom panel. Monomer B shows the interdomain tunnel cavity as transparent orange surface. (B) Key local features and amino acids forming direct hydrogen bonds (dashed lines) to the LL peptide in monomer A. See Figures S7D–S7F for further details of LL-PrfA interactions. (C) Superposition based on residues 2–138 of monomer A of PrfA^WT^ (PDB code 2BEO, yellow), PrfA:LL (PDB code 6HCK, this work, crimson red) and PrfA:GSH (PDB code 5LRR, lilac). Residues 121−138 and 170−195 (HTH-motif) are shown as ribbon diagram. Binding of GSH induces large structural changes in the C-terminal DNA-binding domain of PrfA (residues 139−227), including the formation of water-mediated hydrogen bonds between GSH and Ser177 (dotted lines, water molecules in the PrfA-GSH complex are shown as red spheres). The side chain of Leu1 is sterically hindering the movement of Tyr154 necessary for PrfA activation (red arrow). The distances between Leu1 (crimson) and Tyr154 (lilac) in the superimposed structures are less than 1 Å. See also Figure S7.

Structural evidence for the inhibitory mechanism was obtained through co-crystallization of PrfA with the LL dipeptide. The asymmetric unit of the PrfA-LL complex determined at 2.7 Å resolution contained a biological dimer identical to the previously solved PrfA^WT^ structure (Eiting et al., 2005) (Figure 7A, top). Difference Fourier and Polder electron density maps confirmed the binding of the LL peptide to monomer A only (Figures S7D–S7F; Table S1), as recently seen with synthetic PrfA inhibitors based on ring-fused 2-pyridones (Good et al., 2016). LL is positioned within the interdomain tunnel through hydrogen bonds with the peptide backbone (Figures 7A and 7B). This tunnel was recently identified as the binding site for the GSH cofactor (Hall et al., 2016) (Figure 7A, bottom) and the ring-fused 2-pyridone inhibitory ligands (Good et al., 2016, Kulén et al., 2018). In the PrfA-GSH complex, the backbone torsion angles of the GSH tripeptide are in an extended β strand conformation leading to five main-chain contacts with strands β5 and the turn connecting to β6 (Hall et al., 2016). Combined, these interactions result in the partial collapse of the interdomain tunnel and the positioning of αE from PrfA’s helix-turn-helix (HTH) motif for productive DNA binding (Eiting et al., 2005, Hall et al., 2016) (Figure 7A). Interestingly, the LL peptide is also in an extended conformation and establishes two of the five main-chain contacts made by GSH to β5 (residues Met58–Lys64) (Figure 7B). Despite this and the fact that the side chain of Leu2 occupies the same hydrophobic pocket as the thiol group of the GSH molecule (Hall et al., 2016), wedged between the aromatic residues Phe67 and Tyr126, the position of Leu1 is unique and prevents the collapse of the tunnel needed for PrfA activation. In particular, the 5 Å movement of Tyr154 involved in the intricate network of water-mediated hydrogen bonds connecting the glycine of GSH with Ser177 in the HTH motif (Hall et al., 2016) is sterically hindered by the Leu1 side chain (Figure 7C). Since, in contrast to the LL peptide, GSH has weak affinity for PrfA (K_d_ ≈4 mM, Reniere et al., 2015; undetectable by ITC), our data suggest that the mechanism of peptide-mediated PrfA inhibition involves, at least for some peptides, competitive occupancy of the GSH binding site.

## Discussion

Virulence factors are essential for pathogenesis but a fitness burden in non-infection conditions (Vasanthakrishnan et al., 2015). Pathogens manage this dichotomy through virulence gene regulators, but how they sense the transition into a propitious host habitat remains less well characterized. In this study, we report an environmental regulation mechanism by which *L. monocytogenes* controls the activity of its master virulence switch, PrfA, through the balance of antagonistic effects of inducing and inhibitory peptides scavenged from the medium. Our findings uncover a hitherto undescribed mechanism of direct regulation of a bacterial transcription factor via the oligopeptide composition of the habitat.

Inducing peptides provide Cys residue, which we show is essential for PrfA activation through its key role as rate-limiting GSH precursor (Loi et al., 2015, Lu, 2009). Our results show that PrfA is regulated by the levels of Cys/Cys-peptides in the medium, thus effectively linking the PrfA-GSH system to the environmental conditions. The observed dissociation between the nutritional role of Cys/Cys-peptides and virulence gene activation (Figure S3) is consistent with Cys/Cys-peptides acting as *bona fide* PrfA regulatory signals.

PrfA activation by Cys/Cys-peptides is antagonized by oligopeptides lacking Cys. Based on our data, an abundance of inhibitory peptides explains the weak PrfA-dependent expression levels typically observed in BHI and other complex media (Ripio et al., 1996, Ripio et al., 1997b). Although not a requirement, Leu residues were present in strongly repressing peptides. This was also recently noted by Portman et al. (2017), who independently observed that peptides in the listerial growth medium generally inhibited PrfA. These authors attributed the effect of Leu-containing peptides to inhibition of CodY-mediated *prfA* gene activation (Lobel et al., 2015) in response to either increasing concentrations of branched-chain amino acids (BCAAs) or stringent response dampening upon addition of peptides (Portman et al., 2017). However, our data show that free Leu does not inhibit PrfA (Figure 2H), while the relatively elevated amounts of BCAAs and other amino acids in CDM (in the mM range) are unlikely to trigger a starvation response. Moreover, our experiments with the P14*prfA*^mc^ construct (Figure 5A), which includes the regulatory region targeted by CodY (Lobel et al., 2015), show that the effects of peptides are not due to changes in *prfA* expression but in PrfA protein activity (Figures 5B and 5C). This leaves two possible explanations for the repression mechanism: (1) inhibition via unknown interposed factors or (2) direct interaction with PrfA.

We documented the latter through biophysical studies and co-crystallization of PrfA with inhibitory Leu dipeptide, which located the ligand to the GSH binding site in PrfA’s interdomain tunnel. Strikingly, L-leucylleucine adopts the same extended conformation and position as the γ-glutamylcysteinylglycine tripeptide (Hall et al., 2016), making similar main-chain contacts with PrfA residues (Figure 7B). This is reminiscent of the sequence-independent binding mechanism of the OppA/AppA/DppA receptor proteins of ABC oligopeptide transport systems (Monnet, 2003). The peptide is similarly buried in a cavity between two large protein lobes (Figure 7A), anchored via electrostatic contacts with the invariant α-linked peptide backbone while large water-filled pockets easily accommodate diverse side chains, imposing little binding specificity (Berntsson et al., 2009, Levdikov et al., 2005). The LL/LLL peptides bind to PrfA with μM affinity comparable to that of the ligands accepted by OppA-type peptide-binding proteins (Li et al., 2015). The PrfA interdomain tunnel is spacious enough to accommodate four/six-mer peptides or longer if overhanging outside PrfA. A surface lined with abundant hydrophobic amino acids and also polar groups affords flexible side-chain docking potential, consistent with the ability of peptides of different polarity and composition to cause PrfA inhibition.

Our data provide a working model where the unique set of conformational changes specifically triggered by GSH is hindered by non-specific blockade of PrfA’s GSH binding site by peptides. While activation requires occupancy of the two GSH sites of the PrfA dimer (Hall et al., 2016), non-specific peptide binding to only one monomer (Figure 7A) suffices to alter the correct symmetry of the two HTH motifs, preventing DNA-binding and virulence gene expression. Further work remains to fully characterize the mechanism and dynamics of promiscuous inhibition of PrfA by imported peptides and intermediate breakdown products during their metabolic processing (Figure S8).

Free amino acids are found at low concentrations in soil (≈0.01 to 0.15 μM), whereas oligopeptides are the main organic N source for microbial growth in the environment (Broughton et al., 2015, Farrell et al., 2013). Because Cys is considerably less abundant in proteins compared to other amino acids, soil oligopeptides could be critical, together with other PrfA-repressing environmental signals (temperature ≤ 30°C, plant-derived β-glucosides and other phosphotransferase system (PTS)-transported sugars; de las Heras et al., 2011) in preventing wasteful production of virulence factors outside the host (Figure S8). How then to explain the strong PrfA induction in the peptide-rich cytosol? Interestingly, the Cys content is significantly higher in mammalian proteins (2.3%) than in bacterial or plant proteins (0.5%–1%) (Miseta and Csutora, 2000), which are the main source of organic N in natural ecosystems. These differences may be sufficient to shift the balance of inducing/inhibitory effects of peptides toward PrfA upregulation. Specific cysteine-rich proteins from the host may provide a unique source of PrfA-activating peptides. An example is the Cys-rich miniproteins, which include the chemokines and defensins, secreted by phagocytes or present in cells typically targeted by *L. monocytogenes*, such as macrophages, dendritic cells, and epithelial cells (Lavergne et al., 2012). Adding a layer of complexity, mammalian immunomodulatory peptides may also also cause PrfA inhibition, as illustrated here with the IgG-derived prophagocytic tetrapeptide tuftsin (TKPR) (Wu et al., 2012) (Figure 2F), potentially contributing to virulence fine-tuning during infection. Finally, self-produced and other microbially derived peptides, exemplified by the PplA peptide (ASSLLLVG, Figure 2G), may allow coordinating PrfA regulation according to population density or microbiome conditions (Figure S8).

Based on our *in vitro* functional data, the significantly reduced *actA* induction in Opp^–^ *L. monocytogenes* within macrophages at early/mid time points of infection –comparable to that of the Δ*gshF* mutant– suggests that Cys-peptides contribute to PrfA-GSH system upregulation upon host cell invasion. Although GSH is present intracellularly at high concentrations (1–10 mM) (Banerjee, 2012, Lu, 2009), the GshF dependence of PrfA induction within macrophages (Reniere et al., 2015) (Figure 4A) argues against listerial uptake of host-cell GSH having a main contribution. Free Cys is also unlikely to be sufficient to promote PrfA activation because its intracellular concentrations are normally kept at low (μM), limiting steady-state levels due to its cytotoxicity (Banerjee, 2012, Ohtsu et al., 2010). Interestingly, Opp became progressively dispensable for PrfA activation while the GshF dependence was maintained throughout the infection time course (Figure 4A). Since *L. monocytogenes* is virtually auxotrophic to Cys (Tsai and Hodgson, 2003) (Figure 2D, right), *de novo* bacterial synthesis of GSH obviously depends on an external Cys source. Prolonged infection may lead to gradual depletion of Opp-transported oligopeptides, resulting in critical alteration of the Cys-providing (inducing)/non-Cys-containing (inhibitory) peptide balance, only necessitating the input of relatively minor amounts of free Cys for PrfA induction. Alternatively, other listerial transporters (e.g., dipeptide transporters) may take over the role of Opp in Cys-peptide import, or additional (co)factors may contribute to PrfA activation under Cys/Cys-peptide (GSH)-limiting conditions.

The reported data support a model in which PrfA activity is antagonistically modulated by activating and inhibitory nutritional peptides, with the Opp transport system as a key player upstream of GshF in the PrfA regulation hierarchy (Figure S8). This model reconciles the essentiality of GshF/GSH for PrfA activation (Reniere et al., 2015, Portman et al., 2017) with most known features of listerial virulence regulation, including the contrasting PrfA phenotypes in complex (Ripio et al., 1996, Ripio et al., 1997b) versus chemically defined media (Bohne et al., 1994) or the intriguing “charcoal” effect (Ripio et al., 1996, Ermolaeva et al., 2004). The model provides a unifying framework to interpret how the facultative pathogen *L. monocytogenes* senses niche transitions and adjusts virulence gene expression accordingly.

## STAR★Methods

### Key Resources Table

**Table:** 

REAGENT or RESOURCE	SOURCE	IDENTIFIER
**Antibodies**		

Rabbit polyclonal anti-PrfA	T. Chakraborty / P. Cossart	N/A
Anti-rabbit IgG HRP-linked	Cell Signaling	Cat#7074

**Bacterial and Virus Strains**		

See Table S2	This paper	N/A

**Chemicals, Peptides, and Recombinant Proteins**		

Amberlite XAD4	Sigma-Aldrich	Cat#10357
L-buthionine-(S,R)-sulfoximine (BSO)	Cayman	Cat#14484
Bialaphos	Cayman	Cat#16754
Custom peptides (> 90% purity): ALV, EVFC, EVFL, RGDC, RGDL, TKPC, TKPR, RKDVY, KLLLLK, SQNYPIV, ASSLLLVG	GenScript	N/A
LLL peptide	Sigma-Aldrich	Cat#L0879
LL peptide	Sigma-Aldrich	Cat#L2752
EVF peptide	Sigma-Aldrich	Cat#G3751
Glutathione	Sigma-Aldrich	Cat#G4251

**Critical Commercial Assays**		

RNeasy mini kit	QIAGEN	Cat#74104
AmpliTaq-Gold DNA	Applied Biosystems	Cat#8080245
Deproteinizing sample preparation kit - TCA	Abcam	Cat#ab204708
Intracellular GSH assay kit	Abcam	Cat#ab112132

**Deposited Data**		

3D structure of PrfA-LL peptide complex	Protein Data Bank	PDB ID code 6HCK

**Experimental Models: Cell Lines**		

J774A.1 murine macrophage	ATCC	Cat#TIB-67

**Experimental Models: Mice**		

BALB/c mice	Charles River	Cat#028

**Oligonucleotides**		

See Table S3	This paper	N/A

**Recombinant DNA (plasmids)**		

See Table S2	This paper	N/A

Software and Algorithms		

Mars Data Analysis Software	BMG	https://www.bmglabtech.com/
Prism 7	GraphPad	https://www.graphpad.com/
PHENIX suite	Phenix	http://www.phenix-online.org/
CCP4 suite	Bailey, 1994	http://www.ccp4.ac.uk/
CCP4mg	McNicholas et al., 2011	http://www.ccp4.ac.uk/MG/
Coot	Emsley et al., 2010	https://www2.mrc-lmb.cam.ac.uk/personal/pemsley/coot/
PyMOL v2.2.0	Schrödinger, LLC	https://pymol.org/2/

### Contact for Reagent and Resource Sharing

Further information and requests for resources and reagents should be directed to and will be fulfilled by the Lead Contact, José Vázquez-Boland (v.boland@ed.ac.uk).

### Experimental Model and Subject Details

#### Bacteria, plasmids, culture conditions, chemicals

The strains and plasmids used in this study are shown in Table S2. *Listeria* were routinely grown in porcine BHI (BD-Difco) and *Escherichia coli* in Luria-Bertani (LB) media, with 1% agar for solid cultures. For adsorbent-treated BHI agar, 1% (w/v) Amberlite XAD4 resin (Sigma-Aldrich) or 0.5% (w/v) activated charcoal powder (Merck) was added to the medium prior to autoclaving. For fluid Amberlite XAD4-treated BHI cultures (BHI-Amb), the resin was aseptically removed after autoclaving to avoid interference with optical density (OD) readings. Chemically defined CDM is a modification of the improved minimal medium (IMM) of Phan-Thanh and Gormon (1997), with the following composition: 6.56 g/L KH_2_OP_4_, 30.96 g/L NaHPO_4_ 7H_2_O, 0.41 g/L MgSO_4_, 88 mg/L ferric citrate, 0.1 g/L each of the (L-) amino acids leucine, isoleucine, valine, methionine, arginine, cysteine, histidine and tryptophan, 0.6 g/L L-glutamine, 2.5 mg/L adenine, 0.5 mg/L biotin, 5 mg/L riboflavin, 1 mg/L each of thiamine, pyridoxal, para-aminobenzoic acid, calcium panthothenate and nicotinamide, 5 μg/L thioctic acid and 4.5 g/L glucose. CDM was used freshly prepared from filter-sterilized stock solutions stored at 4°C (except cysteine, glutamine, biotin and ferric citrate solutions, kept at –20°C; and phosphates, MgSO_4_ and glucose, at room temperature). Antibiotic supplements (μg/mL) were as follows (lower values for *Listeria*, others for *E. coli* or both): erythromycin 5 or 250, chloramphenicol 7.5 or 20, spectinomycin 100, carbenicillin 100. All incubations were carried out at 37°C, with 180 rpm orbital shaking for fluid cultures, unless stated otherwise. GSH was kept in reduced state with 2 mM Tris[2-carboxyethyl]phosphine hydrochloride (TCEP) in the stock solution. Chemicals and oligonucleotides were from Sigma-Aldrich unless otherwise indicated.

#### Cell culture

Low passage J774A.1 cells, a female murine macrophage cell line, were maintained in a humidified incubator at 37°C and 5% CO_2_ in DMEM (GIBCO) without antibiotics supplemented with 10% FBS (GIBCO).

#### Mice

Experiments were covered by a Project License granted by the UK Home Office under the 1986 Animals (Scientific Procedures). The Roslin Institute Ethical Review Committee approved this license and the experiments (Project A933). Female, six weeks old BALB/c mice were purchased from Charles River. Mice were group-housed in Level 2 SPF barrier facility at the Roslin Institute, University of Edinburgh (UK), and feed a regular chow diet *ad libitum*.

### Method Details

#### General DNA techniques

PCR was performed with GoTaq DNA polymerase (Promega) for general purposes or high-fidelity PfuUltra II Fusion HS (Agilent) for gene constructs or sequence validation. Plasmid and PCR DNA was purified with QIAprep Plasmid Mini kit and QIAquick PCR purification kit, respectively (QIAGEN). Plasmids were introduced into *L. monocytogenes* by electroporation using a Gene Pulser Xcell apparatus (Bio-Rad) and into *E. coli* by chemical transformation. Restriction enzymes were used according to the manufacturer’s instructions (New England Biolabs). DNA sequences were determined using the Sanger method at Source BioScience (Livingston, UK).

#### Transposon library and screening

A random insertion library was constructed in P14-P_hly-lux_ (wild-type *L. monocytogenes* P14 complemented with a PrfA-regulated bioluminescent gene reporter in the integrative plasmid pPL2*lux*-P_hlyA_ (Bron et al., 2006)) by *himar1* transposon mutagenesis using plasmid pJZ037 (Zemansky et al., 2009). For direct isolation of transposon mutants unable to express PrfA-dependent genes in BHI-Amb, we used the PrfA-regulated virulence gene *hpt* as a “natural” negative selection marker. *hpt* encodes a sugar phosphate (organophosphate) permease that promotes rapid bacterial replication in the host cytosol but which also transports fosfomycin, rendering *L. monocytogenes* susceptible to the antibiotic when PrfA is induced (Scortti et al., 2006). Selection was performed in 150 μg/ml fosfomycin (MIC for P14 in BHIA-Amb ≈12-32 μg/ml) and resistant clones subjected to phenotypic screening and PCR analysis to exclude Tn insertions in *hpt* or *prfA*. The *prfA* gene was also sequenced in all PrfA^–^ mutants with correct PCR patterns for presence of non-synonymous point mutations. Transposition mapping was by colony PCR using relevant oligonucleotides (Table S3).

#### Genetic constructs

Oligonucleotides used to generate PCR fragments for cloning contained suitable restriction site extensions at their 5′ end (Table S3). Complementations were carried out using the pAT29 bifunctional vector with spectinomycin selection (Trieu-Cuot et al., 1990), compatible with the erythromycin resistance marker of the transposable element. For complementation of *oppF*::Tn, the *oppF* gene with its native promoter (P_oppF_, Figure 1A) was PCR-amplified from strain P14 with oligonucleotide primers 21 and 22 and inserted into pAT29’s multicloning site (MCS) (plasmid pAT*oppF*^PoppF^). *oppF* was also placed under the control of the *oppA-F* operon promoter (P_opp_) (Figure 1A) by inserting the corresponding region, amplified using primers 19 and 20, in the adequate orientation into pAT*oppF*^PoppF^ (plasmid pAT*oppF*^Popp^). *gshF* was overexpressed from pAT29 by inserting into the vector’s MCS a PCR segment containing the strong gram-positive promoter *P*δ from the streptococcal pSM19035 plasmid partitioning gene δ (de la Hoz et al., 2000), flanked by SalI and BanHI restriction sites, followed by the *gshF* gene amplified from P14 with primers 25 and 26 (plasmid pAT*gshF*^Pδ^). For insertional mutagenesis of *oppF* and *oppD,* internal PCR fragments to each gene, amplified from P14 with primer pairs 35-36 and 37-38, respectively, were inserted into the bifunctional thermosensitive vector pAULA (Schäferkordt and Chakraborty, 1995), giving the recombinogenic plasmids pAU*oppF* and pAU*oppD*. The same strategy was followed to disable *plcA-prfA* readthrough transcription by insertional disruption of the *plcA* gene (strain P14*prfA*^mc^; primers 39 and 40 were used to generate the internal *plcA* fragment). The in-frame Δ*oppDF* and Δ*gshF* deletion mutants were constructed by allelic exchange. For Δ*oppDF*, primer pairs 31-32 and 33-34 were used to amplify 401-bp and 575-bp fragments corresponding to the first 60 bp of *oppD* and its upstream region and the last 33 bp of *oppF* and its downstream region, respectively. For Δ*gshF*, primer pairs 27-28 and 29-30 were used to amplify 882-bp and 987-bp fragments corresponding to the first 60 bp of *gshF* and upstream region and the last 80 bp of *gshF* and its downstream region, respectively. The amplicons were purified, digested with the appropriate restriction enzymes and inserted into pAULA. After electroporation into *L. monocytogenes*, single and double crossover recombinants were selected by marker selection and confirmed by PCR mapping and DNA sequencing.

#### Growth curves and gene expression analysis

PrfA-dependent gene expression was quantitatively analyzed throughout the *L. monocytogenes* growth curve using a chromosomally integrated *luxABCDE* operon under the control of the PrfA-regulated *hly* promoter (Bron et al., 2006). Overnight bacterial cultures were washed, resuspended in PBS and used to inoculate fresh medium to an initial OD at 600 nm (OD_600_) ≈0.02-0.05. Triplicate 200-μl aliquots were transferred to opaque 96-well plates with clear bottom (ThermoScientific) and OD_600_ and luminescence readings taken every 30 min during incubation in an automated microplate reader (FLUOstar Omega, BMG Labtech). Bioluminescence values were normalized to growth at each time point. RT-qPCR transcription analysis was performed on total RNA samples extracted from mid-exponential *L. monocytogenes* cultures (OD_600_ ≈0.2-0.3 for BHI media) using RNeasy mini kit (QIAGEN) as previously described (Deshayes et al., 2012). The number of transcripts was calculated by interpolation of threshold cycle (C_T_) values of cDNA amplifications in a standard regression curve generated from samples of known DNA concentration. Expession data were normalized by dividing the number of transcripts of the test gene by the geometric mean of the number of transcripts of the reference housekeeping genes *rpoB* and *ldh* (Deshayes et al., 2012). See Table S3 for oligonucleotides used.

#### Characterizaton of PrfA and Opp phenotypes

The PrfA phenotype was examined using three PrfA-regulated genes as natural reporters: *hly* encoding the hemolysin listeriolysin O (LLO), *plcB* encoding the phospholipase C/lecithinase PlcB, and *hpt* encoding the sugar phosphate Hpt permease (Scortti et al., 2006). Hemolytic activity was quantified in U-shaped 96-well microtiter plates by mixing 100 μl two-fold serially diluted culture supernatant (OD_600_ ≈0.2) in 1% dithiothreitol PBS with 100 μl of an 1% suspension of washed sheep erythrocytes in PBS (Ripio et al., 1996). Titers were the reciprocal of the highest dilution where ≥ 50% of hemolysis was visually observed after 90 min incubation at 37°C. PlcB activity was determined by observing the width of the white fatty acid precipitate around the colonies in BHI-based media containing 10% egg yolk suspension (prepared by dispersing one egg yolk in 100 mL of sterile saline) (Ripio et al., 1996). Hpt activity was determined using a sugar acidification test in phenol red base broth (Oxoid) supplemented with 10 mM glucose-1-phosphate (Ripio et al., 1997a). See Figures S1A–S1C. Opp (oligopeptide transport) function was tested by measuring the susceptibility to the toxic tripeptide bialaphos (Borezee et al., 2000). Tests were performed using 6-mm antibiotic assay discs (Whatman) impregnated with 30 μg bialaphos (Cayman Chemical) on CDM plates seeded with 120 μl bacterial culture (OD_600_ ≈0.2), or by monitoring bacterial growth in CDM supplemented with 30 μg/mL bialaphos (see Figure S1E).

#### Western immunoblotting

*L. monocytogenes* bacterial pellets from 10-mL broth cultures collected at OD_600_ ≈0.2-0.3 were washed, resuspended in 500 μL 100 mM Tris, 150 mM NaCl containing Protease Inhibitors Cocktail (Roche) and lysed in Lysin Matrix B tubes with silica beads using a FastPrep homogenizer (MP biomedicals). After centrifuging to remove beads and cell debris, supernatants were collected and the protein concentration determined using a Bradford assay (Sigma-Aldrich). Proteins in bacterial lysates were separated by SDS-PAGE using Bis-Tris Nupage precast gels ran with MOPS buffer (Thermo Fisher), transferred to PVDF membranes, and PrfA protein detected using an anti-PrfA rabbit polyclonal antibody (1:50,000) and anti-rabbit HRP-conjugated secondary antibody (Cell signaling, 1:5,000). Membranes were developed using G-Box chemiluminescent imaging (Syngene), scanned and densitometrically analyzed with Image Studio Lite (LI-COR) using an ≈80-kDa non-specific band as an internal control for normalization.

#### GSH determinations

Total GSH (GSHt = GSH [reduced] + GSSG [oxidized]) was measured in exponentially growing *L. monocytogenes* cells using the GSH assay kit from Abcam. Briefly, bacteria were disrupted by bead-beating as described above, lysates deproteinized using a TCA-based commercial kit (Abcam), and fluorescence determined in an Omega plate reader (BMG).

#### Intracellular infection assays

Intracellular proliferation of *L. monocytogenes* was analyzed in J774A.1 murine macrophages using a standard gentamicin protection assay (Deshayes et al., 2012), with some modifications. Cell monolayers were infected at 10:1 multiplicity for 30 min, washed twice with PBS to remove extracellular bacteria, and incubated in DMEM supplemented with 100 μg/ml gentamicin for 30 min (*t* = 0). In some experiments, J774 cells were deprived of Cys for 4 h and then treated with 200 μM of the GSH-depleting drug L-buthionine-(S,R)-sulfoximine (BSO) (Cayman) 1 h prior to and throughout infection. Intracellular bacterial numbers (IB) were normalized using an “Intracellular growth coefficient” (IGC) at each time point *t* = n respect to the internalized bacteria at *t* = 0 according to the formula: IGC = (IB_*t*n_ — IB_*t*0_) / IB_*t*0_ (Deshayes et al., 2012, Vasanthakrishnan et al., 2015). For intracellular competition assays, monolayers were infected with 1:1 mixes of the competing bacteria and their proportions determined at different time points by PrfA phenotyping on egg-yolk BHI agar (see Figure S1A). The competitive indexes (C.I.) were determined as specified below.

#### Mouse experiments

BALB/c mice were infected via the tail vein with 1.5 × 10^3^ CFU of a ≈1:1 mix of wild-type *L. monocytogenes* and isogenic Δ*oppDF* derivative. After euthanasia at days 0, 3 and 5 after infection, livers and spleens were recovered, homogenized and corresponding bacterial loads determined by plate counting (three mice per group per time point). At least 50 colonies per time point and animal were randomly analyzed to determine the proportion of each bacterial strain by PCR, based on the size of the PCR product (primers ΔoppDF 1 BamHI and ΔoppDF 2 SacI; Table S3). The competitive indexes were calculated using inferred log cfu values with the formula C.I. = (test/reference log cfu ratio at *t* = n)/(test/reference log cfu ratio at *t* = 0).

#### PrfA purification and biophysical assays

Bacterial pellets of IPTG-induced cultures of *E. coli* BL21(pET28a*prfA*^WT^) (Deshayes et al., 2012; Table S2) were resuspended in lysis buffer (50 mM Tris pH 7.5, 300 mM NaCl, 20 mM imidazole) and lysed with an EmulsiFlex homogenizer. After affinity chromatography on a HiTrap HP nickel column in an AKTA system (GE Healthcare), PrfA-containing fractions were pooled together, loaded on a HiTrap Heparin HP column and then on a Superdex 75 Gel Filtration column to remove nucleic acid and protein contaminants. The buffer used for the gel filtration and storage was 50 mM Tris pH 7.5, 300 mM NaCl. Fluorescence-based thermal shift assays were performed in a real-time PCR instrument (Bio-Rad) using 25 μL triplicate samples containing 10 μΜ recombinant PrfA, 5 × SyprOrange (Invitrogen) and 1 mM synthetic peptide. Isothermal Titration Calorimetry (ITC) experiments were performed in a GE MicroCal iTC200 system (GE Healthcare). PrfA was changed to 50 mM Tris pH 7.5, 500 mM NaCl buffer and 100 μΜ of PrfA protein injected into the cell. After the baseline was established for 5-10 min, 2 μL of 250 μM peptide in the same buffer was injected every 60 s into PrfA. Data were analyzed with the inbuilt software of the ITC apparatus using a one-site model. The specific DNA-binding activity of PrfA was measured by biolayer interferometry (BLI) with a FortéBio Octet^RED^ 96 apparatus using a biotinylated double-stranded oligonucleotide containing the P*plcA/hly* PrfA box (Table S3). BLI sensorgrams were determined by dipping streptavidin sensors loaded with the target DNA into wells containing sample buffer (50 mM Tris-Cl pH 7.5 300 mM NaCl, 0.05 Tween 20) to obtain a baseline (60 s), then into wells containing increasing dilutions of PrfA-ligand mixes at 1:100 molar ratio in the same buffer to monitor association (300 s), followed by a dissociation step (300 s). FortéBio data acquisition and analysis v9 software was used to determine binding responses.

#### PrfA-LL co-crystallization, data collection and refinement

For crystallization studies, PrfA was recombinantly expressed in *E. coli* using the pET28a expression vector with a 6-His tag and Tobacco etch virus (TEV) protease cleavage site. The construct encodes the full-length PrfA^WT^ protein with two non-native N-terminal residues (GA) on TEV cleavage. The cleavage product was purified by MonoS 5/5 ion-exchange (GE- Healthcare) with elution at ∼250 mM NaCl in 10 mM Tris pH 7.5, 1 mM DTT, followed by a final size-exclusion chromatography step performed in a HiLoad Superdex 75 16/60 column (GE Healthcare) equilibrated with 20 mM sodium phosphate pH 6.5, 200 mM NaCl. The peak fractions containing PrfA were pooled and concentrated using a Centriprep-10 centrifugal concentrator (Millipore) to a final concentration of 3.5 mg/ml. Purified PrfA (> 95%) in complex with LL was crystallized by the hanging-drop vapor-diffusion method in VDX plates (Hampton Research) at 18°C. Before the crystallization setup, LL was added to the protein solution to a final molar protein-to-ligand ratio of 1:5. Droplets of 4 μL of the protein-LL complex were mixed with 2 μL of reservoir solution consisting of 20% PEG 4000, 100 mM sodium citrate (pH 5.2) and 17% isopropanol. Crystals used for data collection were obtained after 48 h. Diffraction data at −173°C were collected at the ESRF (beamline ID23-2; λ = 0.873 Å). Diffraction images were processed with XDS (Kabsch, 1993) and scaled and merged using AIMLESS from the CCP4 software suite (Bailey, 1994). The structure was determined by molecular replacement with the PHASER program from the PHENIX program suite (Adams et al., 2010) using the high resolution wild-type PrfA structure determined in complex with the inhibitor KSK67 (PDB ID code 6EUT) (Kulén et al., 2018) as the search model. The atomic models were manually built using Coot (Emsley et al., 2010) and refined with PHENIX Refine (Adams et al., 2010). The quality of the electron density map of the ligand was significantly improved in POLDER omit map (Liebschner et al., 2017), and the ligand was modeled with LigandFit with a CC = 0.79 (Figures S7D–S7F) (Terwilliger et al., 2006, Terwilliger et al., 2007). Data collection and refinement statistics are shown in Table S1. Ramachandran outliers are < 0.2%. Figures were prepared with CCP4mg (McNicholas et al., 2011) or PyMOL.

### Quantification and Statistical Analysis

Statistical analyses were performed using GraphPad Prism software. Data with single comparisons were analyzed using two-tailed t test. Data with multiple comparisons were assessed using one-way or two-way ANOVA tests with the appropriate post hoc comparisons, with only relevant comparisons noted on the figures. Figure legends include the exact number of replicates for each experiment and the specific statistical analysis.

### Data Availability

The atomic coordinates and structure factors for the PrfA-LL peptide complex have been deposited in the Protein Data Bank under the ID code PDB: 6HCK.

## Supplemental Information

Document S1. Figures S1–S8 and Tables S1–S3

Document S2. Article plus Supplemental Information
