# Supplementary material for: Control of Bacterial Virulence through the Peptide Signature of the Habitat
Source: Cell Rep. 2019 Feb 12;26(7):1815–1827.e5. doi: 10.1016/j.celrep.2019.01.073 (PMC6389498; doi:10.1016/j.celrep.2019.01.073)
Supplement: Document S1. Figures S1–S8 and Tables S1–S3 [file mmc1.pdf]

Cell Reports, Volume *26*

Supplemental Information

Control of Bacterial Virulence

through the Peptide Signature of the Habitat

Emilia Krypotou, Mariela Scortti, Christin Grundström, Melanie Oelker, Ben F. Luisi, A. Elisabeth Sauer-Eriksson, and José Vázquez-Boland

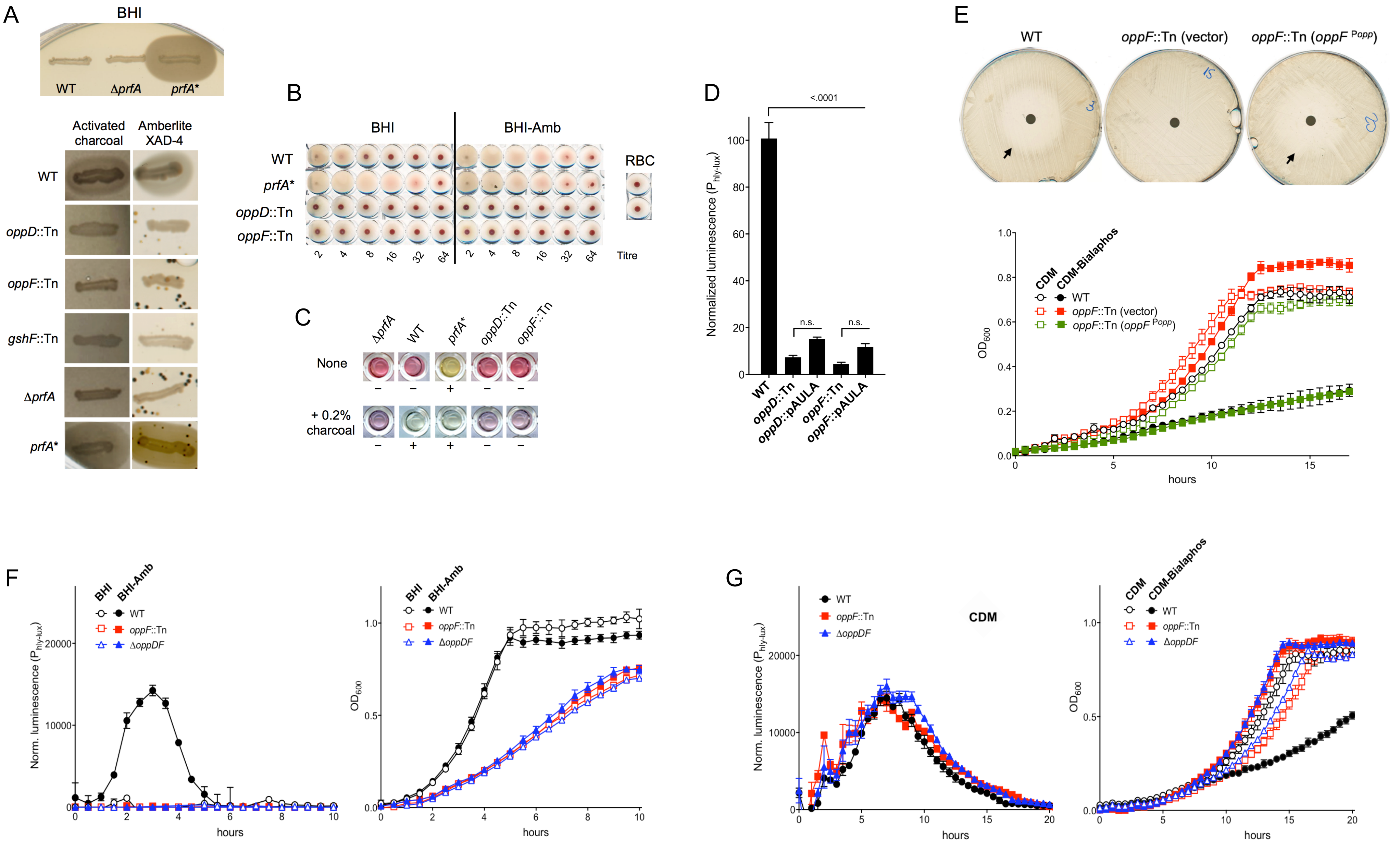

A
BHI
WT
ΔprfA
prfA*
Activated charcoal
Amberlite XAD-4
WT
oppD::Tn
oppF::Tn
gshF::Tn
ΔprfA
prfA*
B
BHI
BHI-Amb
WT
prfA*
oppD::Tn
oppF::Tn
RBC
2 4 8 16 32 64 2 4 8 16 32 64
Titre
C
ΔprfA WT prfA* oppD::Tn oppF::Tn
None
– – + – –
+ 0.2% charcoal
– + + – –
D
<.0001
n.s.
n.s.
Normalized luminescence (Phly-lux)
WT
oppD::Tn
oppD::pAULA
oppF::Tn
oppF::pAULA
E
WT
oppF::Tn (vector)
oppF::Tn (oppF Popp)
CDM
CDM-Bialaphos
WT
oppF::Tn (vector)
oppF::Tn (oppF Popp)
OD600
hours
F
BHI
BHI-Amb
WT
oppF::Tn
ΔoppDF
Norm. luminescence (Phly-lux)
OD600
hours
G
WT
oppF::Tn
ΔoppDF
CDM
CDM-Bialaphos
Norm. luminescence (Phly-lux)
OD600
hours

Fig S1

Figure S1 (Related to Figure 1 and STAR Methods). PrfA phenotype of *opp* (Tn, site-directed and Δ*oppDF*) mutants.

(A-C) $PrfA^{-}$ phenotype of *oppD590*::Tn and *oppF589*:Tn mutants compared to parental wild-type P14 (WT). Markers: PrfA-regulated genes *plcB* (phosphatidyl-choline preferring phospholipase C PlcB), *hly* (hemolysin listeriolysin O [LLO]) and *hpt* (sugar phosphate permease) as reporters (Ripio et al., 1996, 1997ab; Ermolaeva et al., 2004; Scortti et al., 2007; Vasanthakrishnan et al., 2015). P14 derivative with constitutively activated *prfA** allele used as reference. A, PlcB phenotype in egg-yolk BHI agar supplemented with 1% Amberlite XAD4 (visible dots are resin beads) or 0.5% activated charcoal. *opp*::Tn mutants exihibit the same $PlcB^{-}$ phenotype as the *gshF1825*::Tn mutant and control Δ*prfA* strain. Reference phenotypes of *L. monocytogenes* WT, Δ*prfA* and *prfA** in normal BHI in top panel. B, Hly phenotype in BHI and Amberlite XAD4-treated BHI (BHI-Amb) as determined by hemolysin titration in the culture supernatant. RBC, control sheep red blood cell suspension. C, Hpt (organophosphate transport) phenotype in phenol red broth supplemented with 10 mM glucose-1-phosphate, with and without 0.2% activated charcoal. Positive utilization of the sugar phosphate as a result of *hpt* gene activation causes acidification of the medium. This test cannot be carried out with Amberlite XAD4 because the resin adsorbs completely the phenol red pH indicator. Result recorded at 18 h incubation.

(D) Recapitulation of Tn mutant phenotype by targeted disruption of *oppD* or *oppF* by plasmid (pAULA) insertion (see Methods). Maximum normalized $P_{hly\text{-}lux}$ luminescence expressed as percent of wild type (WT). Mean ± SEM of three duplicate experiments. Relevant *P* values (one-way ANOVA) are indicated; n.s., not significant.

(E) Functional characterization of Opp deficiency. Top, bialaphos susceptibility of WT and *oppF*::Tn complemented with empty vector or *oppF* gene in CDM agar. Disks loaded with 50 µg bialaphos, 48 h incubation. Bottom, growth curves of same strains in CDM supplemented with 30 µg/mL bialaphos. Data are mean ± SEM of a representative experiment performed in triplicate.

(F-G) The Δ*oppDF* mutant exhibits the same phenotype as the *opp*::Tn mutants: $PrfA^{-}$ phenotype (F left) and impaired growth (F right) in BHI/BHI-Amb; $PrfA^{+}$ phenotype in CDM (G left); resistance to 30 µg/mL bialaphos (G right). P14Δ*oppDF* used in these experiments was complemented with the $P_{hly\text{-}lux}$ reporter. Mean ± SEM from a representative experiment of three biological repeats each performed in triplicate.

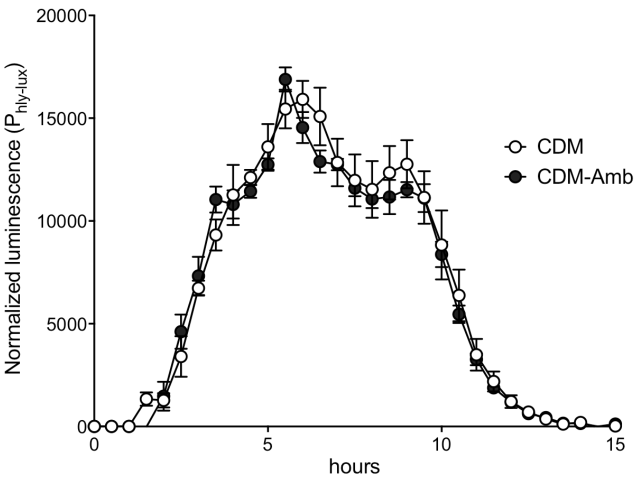

Figure S2 (Related to Figure 1). Lack of effect of Amberlite XAD4 in chemically defined medium.

$PrfA^+$ phenotype of wild-type *L. monocytogenes* remains unaltered in CDM treated with Amberlite XAD4 (CDM-Amb). Normalized expression of $P_{hly\text{-}lux}$ reporter from a representative experiment of three independent biological repeats, each performed in triplicate (mean ± SEM).

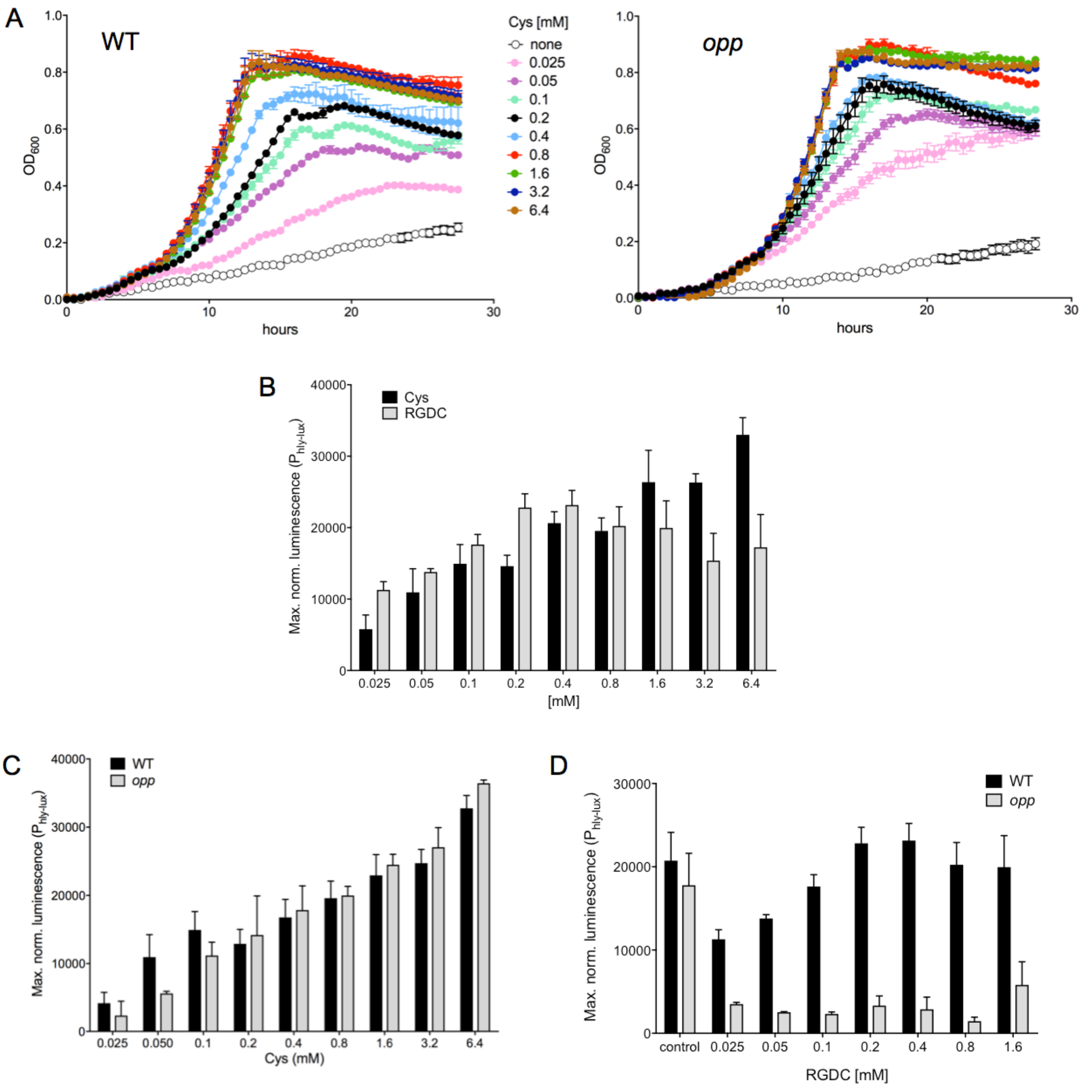

Figure S3 (Related to Figures 2 and 3). L-Cys is required for *L. monocytogenes* growth and PrfA activation.

(A) Cys dependence of *L. monocytogenes* growth. Growth curves of wild-type P14 (WT, left) and isogenic *opp* mutant (right) in Cys-deplete CDM supplemented with increasing concentrations of free Cys. Concentrations ≥12.8 Cys mM inhibit P14 growth. Mean ± SEM of a representative experiment performed in triplicate.

(B) Effect of Cys supplied as free amino acid or as part of a peptide on PrfA activation. $P_{hly\text{-}lux}$ maximum normalized luminescence of WT and *opp* mutant in Cys-deplete CDM supplemented with increasing concentrations of Cys or RGDC peptide. Mean ± SEM of three triplicate experiments.

(C) PrfA activation by Cys amino acid is independent of Opp function. $P_{hly\text{-}lux}$ maximum normalized luminescence of WT and *opp* mutant in Cys-deplete CDM supplemented with increasing concentrations of Cys. Mean ± SEM of three triplicate experiments.

(D) Opp-dependence of PrfA activation by Cys-containing peptide. $P_{hly\text{-}lux}$ maximum normalized luminescence of WT and *opp* mutant in Cys-deplete CDM supplemented with increasing concentrations RGDC peptide. Control is normal CDM (0.8 mM Cys). Mean ± SEM of three triplicate experiments.

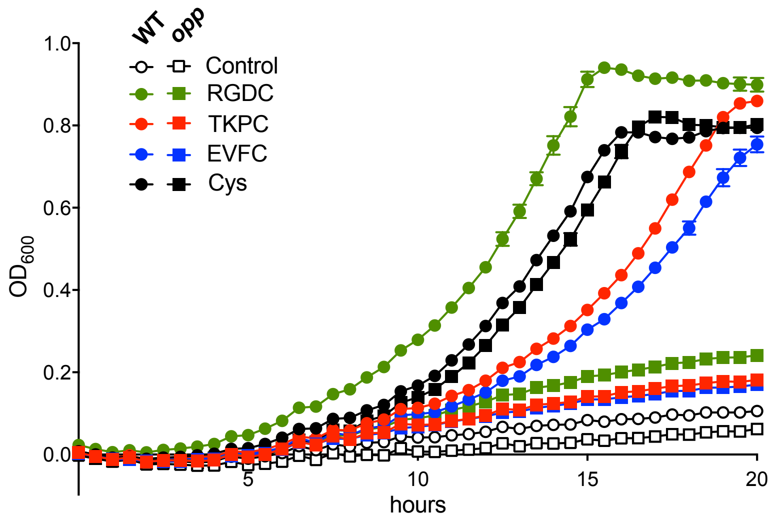

Figure S4 (Related to Figure 2). Opp-dependent utilization of Cys-containing tetrapeptides.

Growth curves of wild-type P14 (WT) and isogenic *opp* mutant in CDM without Cys (control) or supplemented with 1 mM Cys-containing tetrapeptides or free Cys. Mean ± SEM of a representative experiment performed in triplicate.

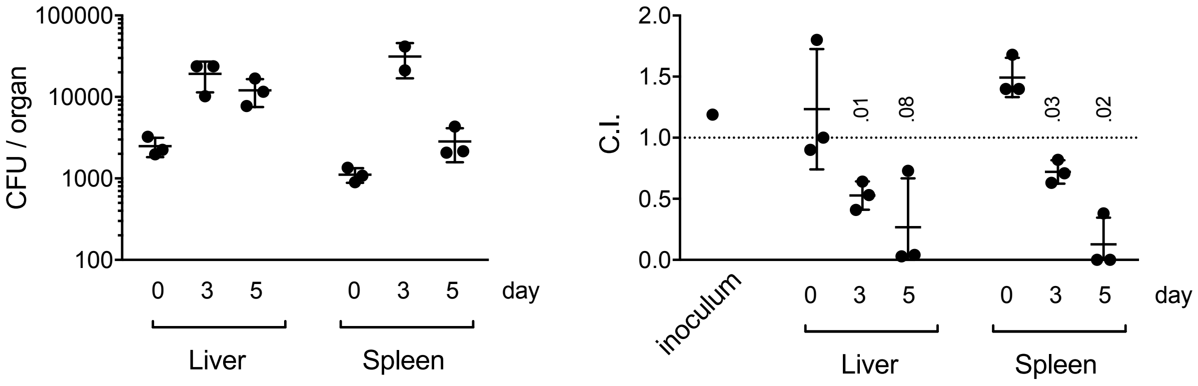

Figure S5 (Related to Figure 4). Virulence attenuation of *L. monocytogenes* Opp^{-} *in vivo*. Competition assay in mouse model of systemic infection. Mice (*n* = 3 per time point) were infected intravenously with a 1:1 mix of wild-type P14 and isogenic Δ*oppDF* (Opp^{-}) mutant (see Fig S1FG) and the competition indexes (C.I.) determined at the indicated time points in the liver and spleens. *t* = 0 corresponds to 1 h after infection. Left, CFU counts per organ. Right, C.I. values. Data are mean ±SEM. C.I. = 1 means equal competing ability, C.I. ≤1 indicates competitive advantage of wild type. Relevant *P* values are indicated (one-sample Student's *t* test, hypothetical value 1, two tails).

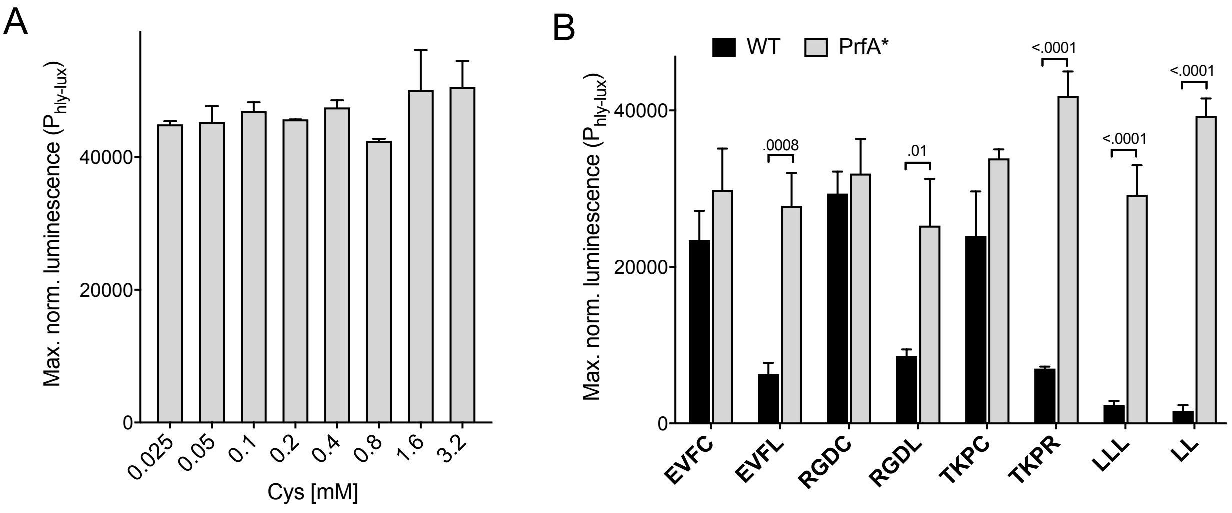

Figure S6 (Related to Figures 4 and 6). ***prfA**** **allele bypasses Cys- and peptide-mediated PrfA modulation.**

(A) $P_{hly-lux}$ maximal normalized luminescence of *L. monocytogenes prfA**^{G145S} in response to increasing concentrations of Cys in CDM.

(B) $P_{hly-lux}$ maximal normalized luminescence of *L. monocytogenes* P14 (WT) and *prfA**^{G145S} derivative in response to 1 mM of the indicated peptides in CDM. Data in grey bars are not significantly different between them.

Data are means ± SEM of three independent experiments performed in triplicate. Statistically significant *P* values are indicated (two-way ANOVA).

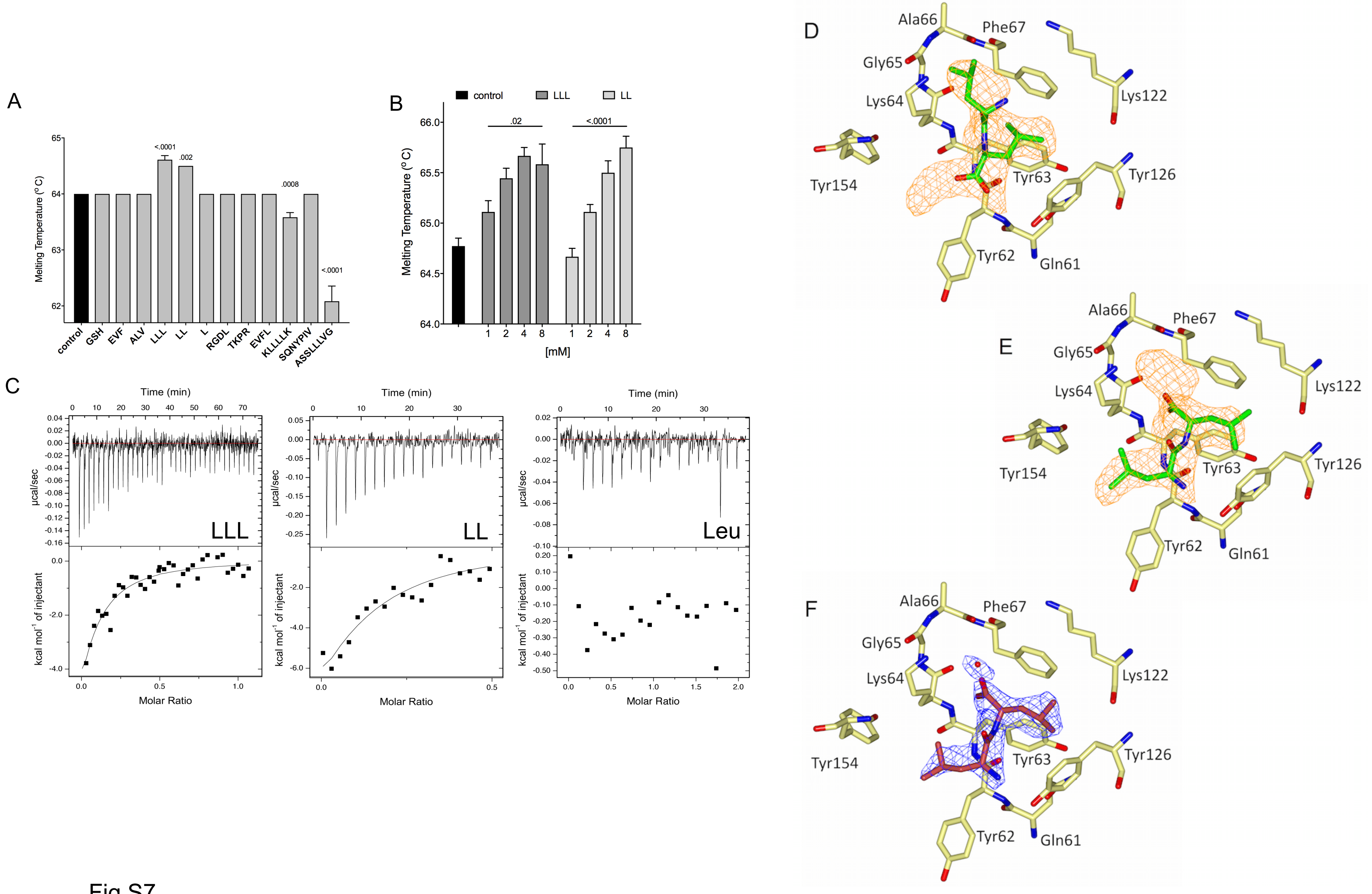
A
B
C
D
E
F
LLL
LL
Leu
Melting Temperature (°C)
control
LLL
LL
[mM]
Time (min)
μcal/sec
kcal mol-1 of injectant
Molar Ratio
Ala66
Phe67
Gly65
Lys64
Lys122
Tyr154
Tyr63
Tyr126
Tyr62
Gln61

Fig S7

Fig S7 (Related to Figures 6 and 7). Biophysical and structural characterization of PrfA-peptide interactions

(A) Thermofluor shift assay with Sypro Orange. Midpoint temperatures of protein-unfolding transition (Tm) determined for PrfA (10 μM) in the presence of 1 mM of the synthetic peptides tested in this study, L-Leu and GSH (control: 300 mM KCl, 100 mM Hepes pH 7.4 buffer alone). Mean ± SEM of three duplicate experiments. Significant P values are indicated (one-way ANOVA). In addition to the reproducible Tm increases with the Leu di- and tripeptides, the inhibitory pPplA pheromone peptide ASSLLLVG (Fig 2G) caused a significant drop in the Tm consistent with PrfA dimer destabilization.

(B) Same as in A with different concentrations of LLL and LL peptides (in 500 mM NaCl, 50mM Tris pH 7.5; control: buffer alone). Mean ± SEM of three duplicate experiments. Statistically significant *P* values are indicated (linear regression).

(C) Representative ITC plots showing binding of LLL and LL peptides to purified PrfA. Top, raw heat emissions against time; bottom, binding isotherms with heat released per mole of injectant plotted against the molar ratio of the two reactants. No binding was detected with 1 mM of free L-Leu (non-inhibitory). Binding constants and thermodynamic parameters for LL peptide: K_a=4.00E4±0.32, N=0.098±0.12, ΔH=-2.11E4±3.098E4 cal/mol, ΔS -49.7 cal/mol/deg; for LLL peptide: K_a =4.12E4±0.23, N=0.047±0.11, ΔH=-2.51E4±6.31E4 cal/mol, ΔS -63.1 cal/mol/deg. The N-values using a 1-site model are likely artifactual due to the hydrophobicity of the LL and LLL peptides, which in solution tend to auto-aggregate. The amount of free peptides able to interact with PrfA was therefore possibly lower than the nominal concentration in the titrations and thus the estimated Kd values (≈25 μM) likely underestimate the affinity.

(D-F) Modelling of the LL peptide in electron density maps of PrfA co-crystallized with the ligand. (D) Polder electron density map (Liebschner et al., 2017) contoured at 4σ (orange) and calculated with the LL peptide (green) positioned as shown in the figure. The Polder map was interpreted by the program as not likely to show the ligand. (E) The LL peptide (green) placed in the Polder map shown in A using LigandFit (Terwilliger et al., 2003, 2007). The program placed all 17 atoms of the LL peptide in the map with overall CC = 0.793. (F) The difference (2|Fo|-|Fc|) electron density map, calculated from the refined PrfA-LL peptide complex and contoured at the RMSD value of the map, is shown in blue covering the LL ligand only. Main contacts of the LL peptide to PrfA (see Fig 6B): Leu1 forms a hydrogen bond to the hydroxyl group of Tyr126 (αC) positioned in the vicinity of the PrfA's DNA-binding helix-turn-helix (HTH) motif. The side chain of Leu1 is positioned in a hydrophobic pocket formed by the side chains of Tyr62 (β5), Lys64 (β5), Leu150 (αD), Tyr154 (αD) and Leu174 (αE). The side

chain of Leu2 is buried in a hydrophobic pocket formed by the aromatic side chains of Tyr63 (β5), Phe67 (β5), Tyr126 (αC), and Trp224 (αH). One oxygen of the carboxyl group of Leu2 forms a hydrogen bond with the backbone nitrogen of Lys64 (β5). The distance to the backbone oxygen of Lys64 (2.8 Å) suggests that it might be protonated. The second carboxyl oxygen forms a hydrogen bond to a water molecule and is positioned ≈4 Å from the α-amino group of Lys64 (β5).

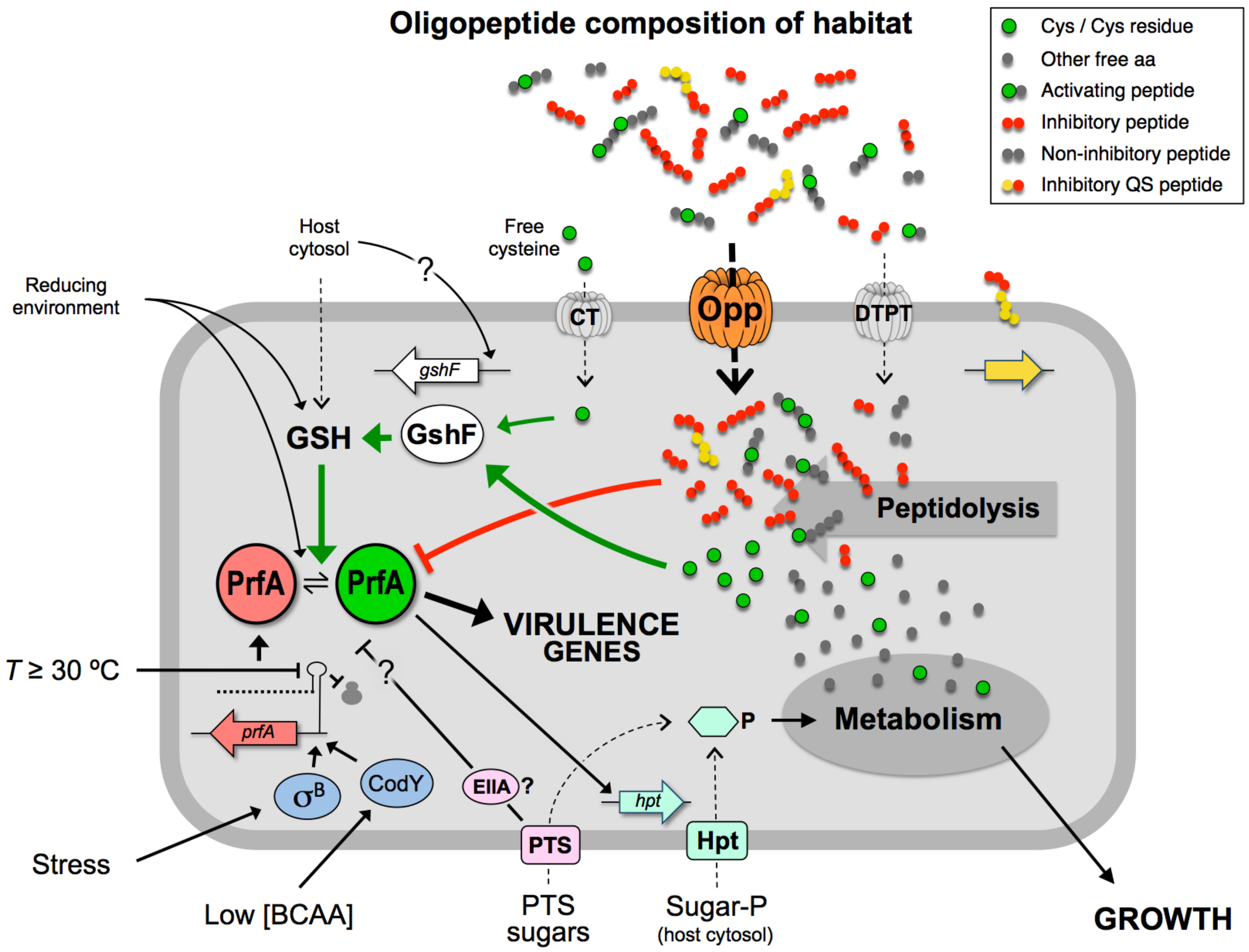

Figure S8 (Related to Figures 1 to 7). Antagonistic PrfA control by Opp imported oligopeptides in *Listeria* virulence gene regulation. PrfA requires GSH synthesized by the listerial GshF enzyme (Gopal et al., 2005) and a reducing environment for full activity *in vivo* in the bacterial system (Reniere et al., 2015; Portman et al., 2017). GSH levels are regulated through the exogenous availability of its rate-limiting precursor, L-cysteine (Cys), imported as a free amino acid (CT indicates Cys transporter) or preferentially in a peptide via the Opp transport system. PrfA induction levels depend primarily on the balance between positive regulation by peptide-derived Cys and promiscuous inhibition by non-Cys-containing peptides (also imported via Opp) and their partial metabolic breakdown products before conversion into amino acids. Smaller peptides can be alternatively imported through dipeptide/tripeptide transporters(s) (DTPT) (Wouters et al., 2005). Outside the host, PrfA inhibition is ensured by environmental peptides in concert with repression by phospho*enol*pyruvate:sugar transport system (PTS)-imported sugars, including glucose and particularly plant-derived β-glucosides such as cellobiose (Scortti et al., 2006; de las Heras et al., 2011), and thermoregulation via an RNA thermoswitch that obstructs *prfA* translation at

temperatures ≤ 30 ºC (Johansson et al. 2002). Bacterially derived peptides, either self-produced (in yellow, "QS" denotes quorum sensing / pheromone peptide) (Ermolaeva et al., 2004) or from the surrounding microbiota, can also cause non-specific PrfA inhibition. Intracellularly, the balance of positive and negative peptide inputs is tilted towards PrfA induction, promoting early virulence gene activation. Within the host, *prfA* translation is enabled at 37 ºC while PTS sugar-mediated PrfA repression is bypassed by utilization of host cell-derived non-PTS carbon sources (sugar phosphates imported via the PrfA-regulated Hpt transporter). PrfA regulation by nutritional peptides constitutes a highly versatile and dynamic environmental gene regulation mechanism directly linking listerial virulence and metabolism. Additional regulatory crosstalk between virulence and metabolism is provided by the global transcription factor CodY, which activates *prfA* gene expression upon sensing low concentrations of branched-chain amino acids (BCAA), particularly isoleucine limitation (Lobel et al. 2015). *prfA* transcription is also activated by the alternative sigma factor SigB during the listerial stress response (Nadon et al., 2002).

Table S1 (Related to Figure 7). Data collection and refinement statistics of crystal structure of PrfA^{WT}-LL complex.

**Data collection**	
Space group	$P2_12_12_1$
Cell dimensions	
a, *b*, *c* (Å)	47.95, 87.39, 116.88
α, β, γ (°)	90.0, 90.0, 90.0
Resolution (Å)	44.4–2.70 (2.80–2.70) *
R_{merge}	0.237 (1.702)
R_{PIM}	0.145 (1.013)
$I / \sigma I$	4.9 (0.6)
CC(1/2)	0.76 (0.37)
Completeness (%)	99.6 (99.6)
Redundancy	6.9 (7.2)
**Refinement**	
Resolution (Å)	44.4-2.70
No. reflections	13956 (1357)
R_{work} / R_{free}	0.209 (0.310)/0.269 (0.291)
No. atoms	
Protein	3777
Ligand/ion	17/2
Water	45
B-factors (Å^2)	
Protein	69.3
Ligand/ion	76.6/44.5
Water	48.2
R.m.s. deviations	
Bond lengths (Å)	0.005
Bond angles (°)	1.04

* One crystal was used for data collection. Values in parentheses are for highest-resolution shell. Estimate of resolution limit is based on CC1/2.

Table S2 (Related to Star Methods). Bacterial strains and plasmids

Strain / plasmid	Description / use	Source (reference)	Internal collection no.
L. monocytogenes			
P14	Serovar 4b wild-type human clinical isolate	Our laboratory (Ripio et al., 1996)	PAM 14
P14*prfA**	Isogenic *prfA**^{G145S} derivative of P14 (also known as P14A)	Our laboratory (Ripio et al., 1996, 1997b)	PAM 50
P14-$P_{hly\text{-}lux}$	P14 with integrated pPL2*lux*-P_{hlyA} plasmid	Our laboratory	PAM 3452
*prfA**-$P_{hly\text{-}lux}$	*prfA**^{G145S} derivative of P14 with pPL2*lux*-P_{hlyA} plasmid	Our laboratory	PAM 3456
oppF::Tn	P14-$P_{hly\text{-}lux}$ himar1 Tn mutant in *oppF* (*oppF*589::Tn, Fig 1A)	This study	PAM 3953
oppD::Tn	P14-$P_{hly\text{-}lux}$ himar1 Tn mutant in *oppD* (*oppD590*::Tn, Fig 1A)	This study	PAM 3948
gshF::Tn	P14-$P_{hly\text{-}lux}$ himar1 Tn mutant in gshF (*gshF1825*::Tn, Fig 1A)	This study	PAM 3955
oppF::Tn (vector)	*oppF*::Tn complemented with pAT29	This study	PAM 4004
oppF::Tn (*oppF*^{Popp})	*oppF*::Tn complemented with pAT29*oppF*^{Popp}	This study	PAM 4073
oppF::Tn (*oppF*^{PoppF})	*oppF*::Tn complemented with pAT29*oppF*^{PoppF}	This study	PAM 4075
opp (*gshF*$^{P\delta}$)	*oppF*::Tn complemented with pAT29*gshF*$^{P\delta}$	This study	PAM 4115
oppD::pAULA	Inactivation of *oppD* by insertion of pAULA in P14-$P_{hly\text{-}lux}$	This study	PAM 3995
oppF::pAULA	Inactivation of *oppF* by insertion of pAULA in P14-$P_{hly\text{-}lux}$	This study	PAM 4057
Δ*oppDF*	In-frame *oppDF* deletion mutant of P14	This study	PAM 4114
*prfA**Δ*oppDF*	In-frame *oppDF* deletion mutant of P14*prfA**	This study	PAM 4120
Δ*prfA*	In-frame *prfA* deletion mutant of P14	Our laboratory (Vega et al., 2004)	PAM 0372
Δ*gshF*	In-frame *gshF* deletion mutant of P14-$P_{hly\text{-}lux}$	This study	PAM 4018
Δ*gshF* (*gshF*$^{P\delta}$)	Δ*gshF* complemented with pAT29*gshF*$^{P\delta}$	This study	PAM 4117
P14*prfA*^{mc}	P14-$P_{hly\text{-}lux}$ expressing monocistronic *prfA* (autoregulation loop disabled, Fig 5A)	This study	PAM 4113
Escherichia coli			
DH5α	Cloning host strain	Our laboratory	PAM 3511
BL21(DE_3) (pET28a*prfA*^{WT})	Production of His-tagged wild-type PrfA protein	Our laboratory (Deshayes et al., 2012)	PAM 3486
**Plasmids**			
pJZ037	Non-integrative plasmid carrying the *himar1* transposon	D. Portnoy (Zemansky et al., 2009)	
pAULA	Thermosensitive shuttle vector for allelic exchange	T. Chakraborty (Schaferkordt and Chakraborty, 1995).	
pAU*oppF*	pAULA inserted with internal *oppF* fragment from P14	This study	

Table S2 (cont.).

Strain / plasmid	Description / use	Source (reference)	Internal collection no.
**Plasmids (cont.)**			
pAU*oppD*	pAULA inserted with internal *oppD* fragment from P14	This study	
pAUΔ*oppDF*	pAULA inserted with recombinogenic construct for *oppDF* deletion	This study	
pAUΔ*gshF*	pAULA inserted with recombinogenic construct for *gshF* deletion	This study	
pAU*plcA*	pAULA inserted with internal sequence of *plcA* for construction of monocistronic *prfA* strain	This study	
pAT29	Complementation vector with spectinomycin resistance marker	Institut Pasteur (Trieu-Cuot et al., 1990)	
pAT*oppF*^{Popp}	PAT29 carrying *oppF* and the *opp* operon promoter from P14	This study	
pAT*oppF*^{PoppF}	PAT29 carrying *oppF* and its native promoter from P14	This study	
pAT*gshF*$^{P\delta}$	PAT29 carrying *gshF* from P14 and the constitutive promoter Pδ	This study	
pPL2*lux*-P_{hlyA}	*luxABCDE* under control of P*hly* promoter inserted in pPL2 integrative plasmid	C. Hill (Bron et al., 2006)	

Table S3 (Related to Star Methods). Main oligonucleotides and probes used in the study. Relevant restriction sites are in bold

Oligonucleotide			
**No.**	**Name**	**Sequence**	**Use**
1	qPCR-*actA* F	AAGAAATTGATCGCCTAGCTGATT	qPCR of *actA*
2	qPCR-*actA* R	GTAAAAAACCCGCATTTCTTGAGT	
3	qPCR-*actA* P	FAM-TTTCCTGTTCCTCTATCTCT-TAMRA	
4	qPCR-*gshF* F	GCGGAACAGACCATTTGAAAC	qPCR of *gshF*
5	qPCR-*gshF* R	GGTATGCTGTCCCAAGAAAGG	
6	qPCR-*gshF* P	FAM-TGGTCCAGAAGAAACGCTCATGCT-TAMRA	
7	qPCR-*ldh* F	ATGCTCGTAACGTCCATGGTT	qPCR of *ldh*
8	qPCR-*ldh* R	GCTCCATGCTGGGAATTCTG	
9	qPCR-*ldh* P	FAM-CATCCTTGGCGAACACGGCGA-TAMRA	
10	qPCR-*rpoB* F	TGGTTCTTAGATGAAGGGCTACGT	qPCR of *rpoB*
11	qPCR-*rpoB* R	ACCCGCAAAATCCTCAATTG	
12	qPCR-*rpoB* P	FAM-AATATCGCGGAACATCT-MGB-NFQ	
13	*prfA* locus F	CTTGGCGAAGCAATCGT	External to *prfA* to check integrity of locus
14	*prfA* locus R	GGAATGCAGATGCATCCTTTGC	
15	*hpt* locus F	CGATCGAATTATCGTTGTAA	External to *hpt* to check integrity of locus
16	*hpt* locus R	GAGCAGCGATTACTTTCATC	
17	*pδ* prom SalI F	CGCG**GTCGAC**AATCACAAATCACTTATCACAAATC	Cloning of Pδ promoter in pAT29
18	*pδ* prom BamHI R	CG**GGATCC**CATTCTTTTTTTGTATAGTAATATT	
19	*opp* prom SalI F	GCGC**GTCGAC**GGTTATGTTTTCTGAAAACTTAATCAAAAGG	Cloning of *opp* promoter in pAT29
20	*opp* prom BamHI R	GCG**GGATCC**TAAGTAGACCTCCCTTTTTTATTTTTCTG	
21	*oppF* BamHI F	GCG**GGATCC**GACGCATTTGCTGCGCG	Cloning of *oppF* in pAT29
22	*oppF* SacI R	GCGC**GAGCTC**GAAAAAAATAGCTTTTTTATGAGAATTTCATGAG	
25	*gshF* BamHI F	GCG**GGATCC**CGTCCATCTTTTTAGTTAAGTTCCG	Cloning of *gshF* in pAT29
26	*gshF* SacI R	GCGC**GAGCTC**GCTACGAAACTAGTAGCTGGG	
27	Δ*gshF* 1 BamHI	GCG**GGATCC**CACCGCATCGGTCCGCA	Deletion of *gshF*
28	Δ*gshF* 2 SalI	GCG**GTCGAC**CTTCCGGAGCTTAGGGTC	
29	Δ*gshF* 3 SalI	GCG**GTCGAC**GCGATGCACATGCACTGC	
30	Δ*gshF* 4 XmaI	CC**CCCGGG**GGACGTCATGCGGAGGGAGAT	

Table S3 (cont.)

Oligonucleotide No.	Oligonucleotide Name	Sequence	Use
31	Δ*oppDF* 1 BamHI	GC**GGATCC**TGGCTTCCATGACTTTAGGG	Deletion of *oppDF*
32	Δ*oppDF* 2 SalI	GCG**GTCGAC**TTCACCGGCATATGTGTGG	
33	Δ*oppDF* 3 SalI	GCG**GTCGAC**GCTAAGCTAACTGCTGAAGC	
34	Δ*oppDF* 4 SacI	GCGC**GAGCTC**GCCTTCGATTTGCTTGGTGG	
35	*oppF* ins BamHI F	GCG**GGATCC**CGGTAAAGATGTGCACGCACG	Insertional mutagenesis of *oppF*
36	*oppF* ins SalI R	GCG**GTCGAC**GCGCGAAATTGCTCCTGG	
37	*oppD* ins BamHI F	GCG**GGATCC**GCGTTTACTTCCAGAAGGTAACTC	Insertional mutagenesis of *oppD*
38	*oppD* ins SalI R	GCG**GTCGAC**CAGGTGGCGTACCAGGAATAAC	
39	*plcA ins* BamHI F	GCG**GGATCC**TACTCGGACCATTGTAGTCATC	Insertional disruption of *plcA-prfA* readthrough transcription
40	*plcA ins* SacI R	CCC**GAGCTC**GTCCGCTCTACCTGACAC	
41	TN 1	GCTTCCAAGGAGCTAAAGAGGTCCCTAGCGCC	Amplification and mapping of the transposon insertion
42	TN 2	CGGGGAATTTGTATCGATAAGGAATAGATTTAAAAATTTCGCTGTTATTTTG	
43	ARB 1	GGCCACGCGTCGACTAGTACNNNNNNNNNNCTTCT	
44	ARB 2	GGCCACGCGTCGACTAGTAC	
45	TN Seq	ACAATAAGGATAAATTTGAATACTAGTCTCGAGTGGGG	Sequencing of the transposition insertion
46	Phlybox-F	Biotin-TEG-TGTCCCTTTATCGTCGTTAACAAATGTTAATGCCTCGACA	DNA-binding experiments (PrfA box of P*hly* / P*plcA* underlined)
47	Phlybox-R	Biotin-TEG-TGTCGAGGCATTAACATTTGTTAACGACGATAAAGGGACA	
